# Bioinspired Ruthenium Arene Complex with Pseudo-Vacant Coordination Sites as Efficient Small-Molecular Antioxidant Enzyme Mimics for Preventing Vascular Restenosis

**DOI:** 10.34133/research.1353

**Published:** 2026-07-03

**Authors:** Haojie Xu, Yichen Cai, Wei Geng, Zhenyu Xing, Jiayan Zheng, Xiaolin Wang, Yang Gao, Qiu Chen, Li Qiu, Chong Cheng

**Affiliations:** ^1^ Department of Medical Ultrasound, West China Hospital, Sichuan University, Chengdu 610041, China.; ^2^ Department of Endocrinology, Hospital of Chengdu University of Traditional Chinese Medicine, Chengdu 610072, China.; ^3^ Shanghai Sixth People's Hospital Affiliated to Shanghai Jiao Tong University School of Medicine, Shanghai 200233, China.; ^4^ College of Polymer Science and Engineering, State Key Laboratory of Advanced Polymer Materials, Sichuan University, Chengdu 610065, China.; ^5^ Department of Endodontics, Department of Orthodontics, State Key Laboratory of Oral Diseases & National Clinical Research Center for Oral Diseases, West China Hospital of Stomatology, Sichuan University, Chengdu 610041, China.; ^6^ School of Pharmacy and State Key Laboratory of Quality Research in Chinese Medicine, Macau University of Science and Technology, Macao 999078 China.

## Abstract

With the ongoing progress in coronary interventional therapy, the progression of atherosclerosis is no longer solely governed by its natural course. Postprocedural neointimal hyperplasia and plaque formation markedly increase the risk of cardiovascular events, where chronic, unresolved inflammation and the overproduction of reactive oxygen species (ROS) play a critical role in driving plaque development. Drawing inspiration from the reaction mechanisms of intracellular antioxidant defense systems, we report the de novo design of a ruthenium arene complex (RuC_6_H_6_) with pseudo-vacant coordination sites, which serves as efficient small-molecular antioxidant enzyme mimics for biocatalytic ROS elimination and the attenuation of vascular restenosis. Our findings reveal that the ligands in RuC_6_H_6_ undergo rapid hydrolysis during the reaction and chloride ligands are replaced by H_2_O_2_, thereby generating abundant reactive sites and conferring the complex with efficient, broad-spectrum, and stable ROS-scavenging properties. When encapsulated in liposomes (Lipid-Ru), the complex effectively reprogrammed macrophages from the M1 phenotype, diminished the secretion of inflammatory cytokines, and suppressed foam cell formation. Moreover, the Lipid-Ru administration led to a significant reduction in smooth muscle cell migration and neointimal area, ultimately alleviating vascular restenosis. This class of structurally tunable arene ruthenium complexes thus provides a promising strategy for designing biomimetic antioxidant reagents, holding considerable potential for the treatment of diverse inflammation-associated diseases.

## Introduction

Atherosclerosis and its complications, including myocardial infarction and stroke resulting from arterial stenosis, present a substantial global health burden and are leading causes of worldwide morbidity and mortality [1]. Coronary artery stenosis represents a critical manifestation of atherosclerotic disease. Current clinical management relies primarily on pharmacotherapy, balloon angioplasty [2], and percutaneous coronary intervention [3]. While these treatments yield favorable outcomes, adverse sequelae following these procedures remain a concern. Studies indicate that restenosis underlies 30% to 40% of long-term failures after coronary revascularization. This process triggers excessive production of pathological reactive oxygen species (ROS) and promotes vascular inflammation, contributing to postoperative pathological remodeling. In response to these challenges, considerable progress has been made in the development of interventional devices, including bioresorbable stents [4], drug-eluting stents [5], and drug-coated balloons [6]. However, the clinical benefits of these technologies have been constrained by persistent risks of restenosis and issues related to drug stability [7–9]. Inflammatory response and oxidative stress are critical contributors to restenosis following balloon angioplasty. During balloon angioplasty, the procedure induces localized endothelial cell loss and triggers aberrant activation of smooth muscle cells (SMCs), which then migrate to the injured site [10,11]. The damaged vascular region exhibits heightened secretion of inflammatory factors and recruitment of immune cells such as macrophages. In the context of underlying conditions such as hyperlipidemia, these inflammatory responses synergistically drive the restenosis process.

In the human body, endogenous antioxidant enzymes such as catalase (CAT) and superoxide dismutase (SOD) play a fundamental role in maintaining oxidative homeostasis under physiological conditions [12–14]. Nevertheless, therapeutic approaches based on natural enzymes are constrained by inherent limitations, including a short circulating half-life, low cellular permeability, and potential immunogenicity, which limit their direct application in treating inflammation-induced tissue damage [15]. In response, chemists and materials scientists have sought to mimic natural antioxidant enzymes by designing synthetic biocatalytic materials for a range of antioxidant therapies [16–18]. Recent efforts have focused on developing antioxidant nanostructures derived from metal hydroxides [19], nanocarbons [20], metal oxides [21], and other inorganic systems, aiming to eliminate ROS and mitigate atherosclerosis. Although evidently progress has been made in these materials, several issues, such as poorly defined active sites and potential biosafety concerns, may complicate rational design and precise synthesis based on natural enzymatic centers, ultimately limiting systematic improvements in catalytic performance and hindering further clinical application. To address these challenges, researchers have turned to polymeric and organometallic complexes as functional mimics of antioxidant enzymes, such as polyporphyrin [22], polyphthalocyanine [23], and those complexes incorporating porphyrin, salphen, or macrocyclic aza-ligands [24]. These advances highlight the promise of organic scaffolds as a platform for the rational design of high-performance artificial enzymes.

The accessibility of the active site is a crucial factor governing the catalytic reactivity of natural enzymes. In enzymatic reactions, substrates typically enter the hydrophobic pocket before coordinating to the central metal ion [25]. In many natural enzymes, the metal active site is coordinated to a water molecule, which is stabilized by a hydrogen-bonding network with surrounding amino acid residues. The displacement of this water molecule by the substrate often constitutes a key step in enzymatic synthesis or transfer reactions [26,27]. Recently, our group has highlighted the promise of ruthenium, an iron-group metal with favorable biocompatibility and distinctive biocatalytic properties. Compared to iron, ruthenium possesses more *d* electrons, accessible unoccupied orbitals, and enhanced redox stability, contributing to its superior catalytic performance [28,29]. These characteristics make ruthenium-based organometallic complexes with high reactivity and accessibility compelling targets for biomimetic design. Furthermore, recent studies have shown that ruthenium arene complexes represent a versatile class of catalysts widely employed in diverse organic transformations, including hydrogenation, esterification, olefin metathesis, and cycloaddition reactions [30]. In these complexes, the ruthenium center is coordinated by an arene ring alongside mono- or bidentate ligands. The η^n^-bonded arene ligand is relatively inert toward substitution. This stabilizes and protects the metal center, thereby preventing rapid oxidation from Ru(II) to Ru(III) [30,31]. Moreover, the coordination sites that trans to the arene ligand can be readily modified, enabling substantial structural and functional tunability [30]. Owing to their well-defined active sites, tunable catalytic efficiency, and synthetic versatility, molecular ruthenium arene complexes may serve as ideal candidates for the development of high-performance artificial enzymes, while the well-defined structures and bioactivities remain unexplored.

**Fig. 1.**
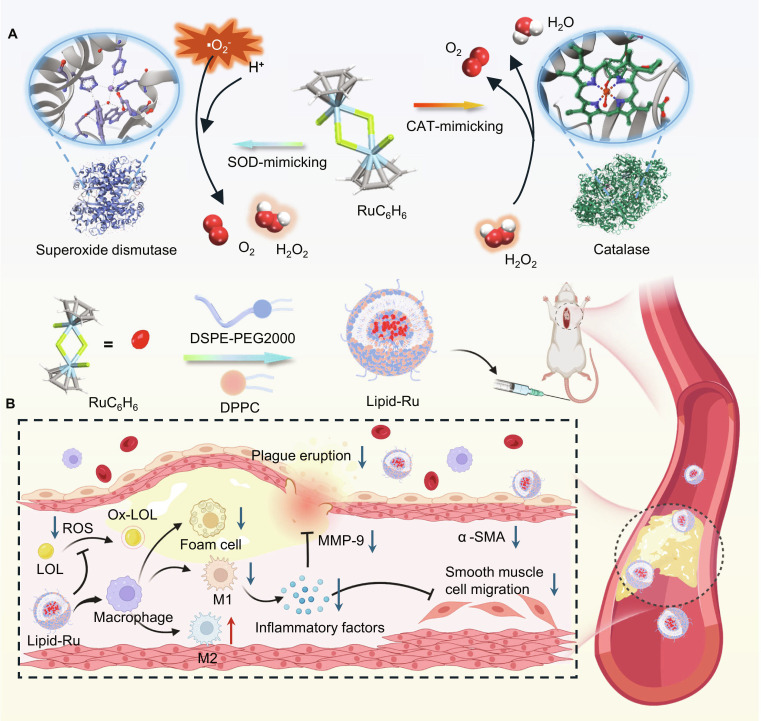
Design of ruthenium arene complex (RuC_6_H_6_) with pseudo-vacant coordination sites for efficient biocatalytic ROS elimination and attenuation of vascular restenosis. (A) Bioinspired design of RuC_6_H_6_ as a small-molecular antioxidant enzyme mimic and the corresponding development of the liposome-loaded nanoparticle (Lipid-Ru). (B) Illustration of the therapeutic mechanisms of Lipid-Ru for post-angioplasty restenosis.

In this work, we report the bioinspired design of a ruthenium arene complex (RuC_6_H_6_) with pseudo-vacant coordination sites, serving as small-molecular antioxidant enzyme mimics for efficient biocatalytic ROS elimination and attenuation of vascular restenosis. Our findings reveal that the RuC_6_H_6_-based antioxidant enzyme mimic exhibits superior radical scavenging performance, closely mimicking the multifunctional activities of endogenous antioxidant enzymes, such as CAT and SOD. Further investigation into CAT-like pathways revealed that the presence of a labile chloride ligand at the pseudo-vacant site facilitates the coordination of H_2_O_2_ to the ruthenium center, thereby promoting catalytic turnover. To improve bioavailability and extend circulation half-life while leveraging the enhanced permeability and retention (EPR) effect for targeted accumulation at vascular endothelial injury lesions, the selected complex was encapsulated within polyethylene glycol (PEG)-modified liposomes, yielding Lipid-Ru [32,33], which exhibited excellent stability and sustained release characteristics (Fig. 1A). Within the lesion, Lipid-Ru effectively scavenges ROS, attenuates ROS-driven inflammatory responses, reverses M1 macrophage polarization, and reduces foam cell formation. Moreover, Lipid-Ru significantly curtails oxidative stress-induced SMC migration and plaque destabilization. It down-regulates key inflammatory genes and signaling pathways, thereby suppressing processes linked to smooth muscle proliferation and plaque vulnerability in the aortic wall (Fig. 1B). In summary, this study establishes a rational design strategy for metal–organic complexes that mimic antioxidant enzymes, effectively reducing the risk of vascular restenosis following balloon angioplasty. These results suggest the considerable potential of structurally tunable ruthenium arene complexes as bioinspired catalytic therapeutics for a spectrum of inflammation-associated pathologies, including post-angioplasty restenosis, arthritis, diabetic wounds, and inflammatory bowel disease.

## Results and Discussion

### Structural characterization and antioxidant enzyme-like activity evaluation

**Fig. 2.**
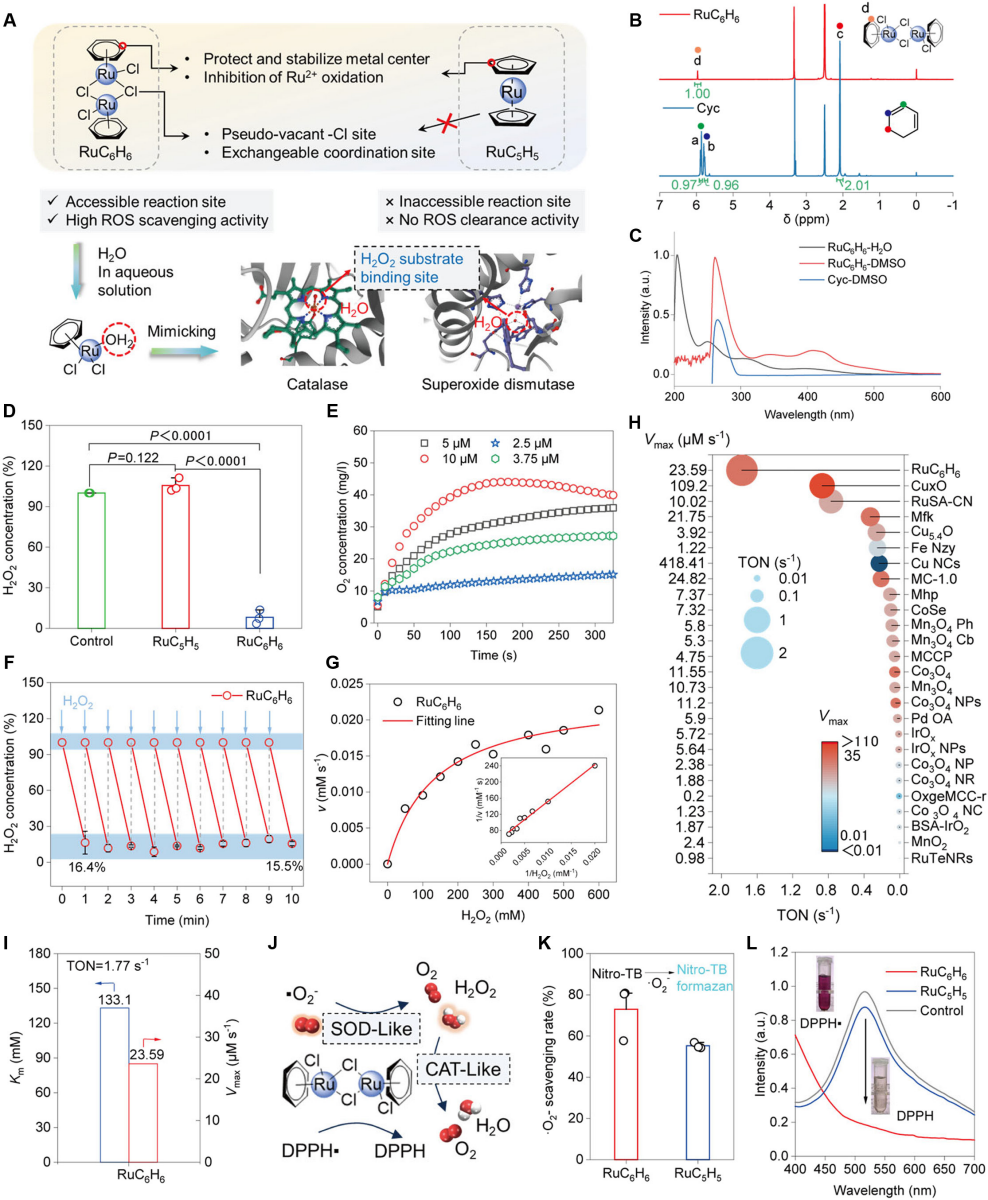
ROS-elimination activities of arene ruthenium complexes. (A) Schematic mechanism illustration of the CAT- and SOD-mimetic activities of RuC_6_H_6_. (B) ^1^H-NMR spectrum of 1,3-cyclohexadiene and RuC_6_H_6_. (C) UV–Vis spectrum of 1,3-cyclohexadiene and RuC_6_H_6_ in H_2_O and dimethyl sulfoxide (DMSO). (D) The H_2_O_2_ elimination activities at 5 min (*n* = 3 independent experiments, data are presented as mean ± SD). (E) The dynamic O_2_ generation property of RuC_6_H_6_ at different times. (F) Cyclic catalytic ability of RuC_6_H_6_ after adding H_2_O_2_ (*n* = 3 independent experiments, data are presented as mean ± SD). (G) Typical Michaelis–Menten curves and double-reciprocal plots for determining the kinetic constants of RuC_6_H_6_ with H_2_O_2_ as substrate. (H) Comparison and analysis of the TON and *V*_max_ values with previously published enzyme-mimetic materials. (I) *V*_max_, *K*_m_, and TON values of RuC_6_H_6_. (J) Schematic diagram of the cascaded catalysis of SOD-like, CAT-like, and DPPH• scavenging reactions. (K) SOD-like property of RuC_5_H_5_ and RuC_6_H_6_ for scavenging of •O_2_^−^ and (L) DPPH• scavenging activity of RuC_5_H_5_ and RuC_6_H_6_ (*n* = 3 independent experiments, data are presented as mean ± SD). *V*_0_ is the initial velocity, *V*_max_ is the maximal reaction velocity, *K*_m_ is the Michaelis constant, and TON is the turnover number. Experiments were repeated independently (D to I) 3 times with similar results. In (C) and (L), a.u. indicates the arbitrary units. *P* values are assessed by a 2-sided Student *t* test.

To systematically evaluate the H_2_O_2_ decomposition capacity of ruthenium arene complexes, we synthesize and compare 2 fundamental precursor materials, [(η_6_-C_6_H_6_)RuCl_2_]_2_ (RuC_6_H_6_) and [(η_5_-C_5_H_5_)_2_Ru] (RuC_5_H_5_), which serve as common synthetic starting points for most arene ruthenium complexes (Fig. 2A). The RuC_6_H_6_ dimer was synthesized following a previously reported procedure [34] and thoroughly characterized by nuclear magnetic resonance (NMR), ultraviolet-visible (UV–Vis), and Fourier transform infrared (FTIR) spectroscopy (Fig. 2B and C and Figs. S1 to S3). RuC_5_H_5_ was obtained from a commercial fine-chemical supplier and used as received. The H_2_O_2_ decomposition activity of both complexes was assessed using a peroxide–titanium complex assay [35,36] in phosphate-buffered saline (PBS) buffer (0.01 M, pH 7.4) to simulate the H_2_O_2_ scavenging performance of RuC_6_H_6_ in a physiological environment. Notably, RuC_6_H_6_ exhibited efficient H_2_O_2_ elimination, whereas RuC_5_H_5_ showed minimal decomposition activity under identical conditions (Fig. 2D and Fig. S4). The catalytic performance of RuC_6_H_6_ was also found to be concentration-dependent (Fig. S5).

To further characterize the reaction products, we employed a dissolved oxygen meter to monitor O_2_ generation. These measurements confirmed that RuC_6_H_6_ efficiently decomposes H_2_O_2_ to produce oxygen, with O_2_ yield increasing as a function of RuC_6_H_6_ concentration (Fig. 2E). Moreover, RuC_6_H_6_ exhibited excellent catalytic durability, maintaining approximately 90% H_2_O_2_ elimination efficiency over 10 successive reaction cycles (Fig. 2F) and sustaining high CAT activity over a period of 5 days (Fig. S6). We also investigated the influence of pH on the CAT-like activity of RuC_6_H_6_, observing enhanced H_2_O_2_ decomposition with increasing pH (Fig. S7). This behavior is likely attributable to higher H^+^ concentrations competing with H_2_O_2_ for binding at the Ru–O active site, thereby attenuating catalytic efficiency [37]. Reaction kinetics were subsequently analyzed across H_2_O_2_ concentrations ranging from 50 to 600 mM. Initial rates measured at varying substrate concentrations were well-described by Michaelis–Menten kinetics, as evidenced by linear double-reciprocal plotting (Fig. S8). From this analysis, the Michaelis constant (*K*_m_) was determined to be 133.1 mM, the maximum reaction velocity (*V*_max_) was 23.59 μM·s^−1^, and the turnover number (TON) was 1.77 s^−1^ (Fig. 2G to I). Notably, RuC_6_H_6_ demonstrates highly favorable catalytic metrics relative to previously reported CAT-mimetics, including metal oxides, nanoparticles, single-atom materials, and metal–organic frameworks (Table S1). Collectively, these results establish RuC_6_H_6_ as an efficient and recyclable catalyst for the decomposition of H_2_O_2_ into H_2_O and O_2_.

We next evaluated the capacity of arene ruthenium complexes to scavenge additional ROS (Fig. 2J). First, we evaluated the total radical scavenging capacity of the material using the ABTS [2,2′-azino-bis(3-ethylbenzothiazoline-6-sulfonic acid)] radical scavenging assay. The results showed that RuC_6_H_6_ demonstrated superior ABTS^+•^ radical scavenging activity, which was approximately 1.7-fold higher than that of RuC_5_H_5_ (Fig. S9). In biological systems, SOD plays a critical role in ROS elimination by catalyzing the conversion of •O_2_^−^ to O_2_ and H_2_O_2_. Using the nitrotetrazolium blue chloride assay [38], we found that RuC_6_H_6_ exhibits pronounced SOD-like activity, achieving an •O_2_^−^ scavenging rate of 80.74%, which is substantially higher than the 55.28% observed for RuC_5_H_5_ (Fig. 2K). Furthermore, RuC_6_H_6_ displayed specific scavenging ability toward the 1,1-diphenyl-2-picrylhydrazyl radical (DPPH•), a stable radical widely used to simulate oxidative stress conditions and assess antioxidant capacity (Fig. 2L). In contrast, RuC_5_H_5_ showed negligible activity against DPPH•. Together, these results demonstrate that RuC_6_H_6_ possesses broad-spectrum ROS-scavenging capability, with particularly efficient CAT-like activity when compared to other arene ruthenium complexes (RuC_5_H_5_).

### Experimental and theoretical study on antioxidase-like activities

**Fig. 3.**
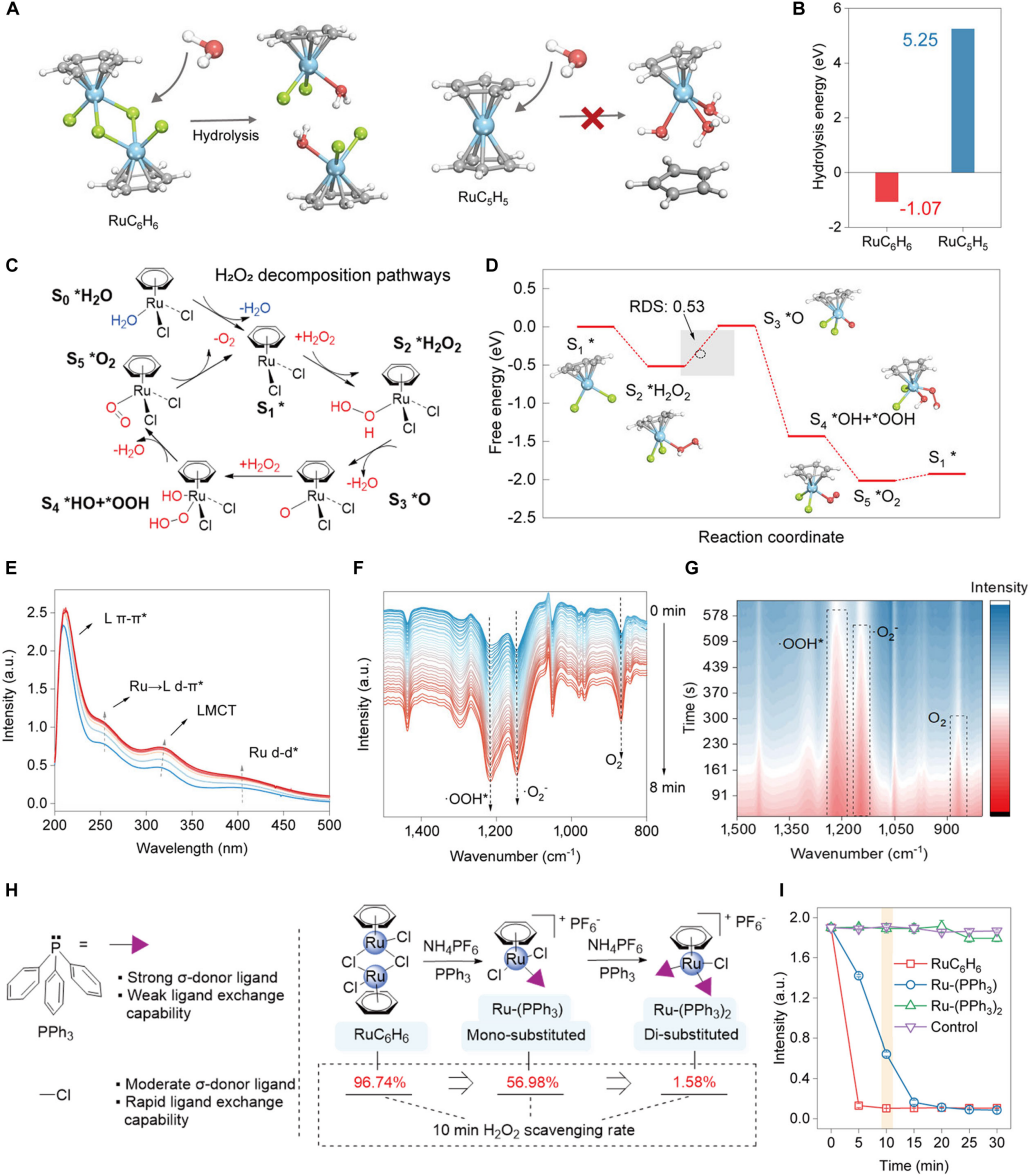
Theoretical analysis of CAT-like catalytic pathways. (A) Comparison of the hydrolysis process between RuC_6_H_6_ and RuC_5_H_5_, and (B) the corresponding hydrolysis energy. (C) Calculated CAT-like catalytic pathways, and (D) corresponding free energy profiles. (E) Time-dependent UV–Vis spectrum during catalytic H_2_O_2_ decomposition. (F) In situ FTIR spectrum and (G) the corresponding contour plot of RuC_6_H_6_ for H_2_O_2_ decomposition. (H) Preparation protocol of replacing chlorine with PPh_3_ and 10 min scavenging rate of H_2_O_2_. (I) Time-dependent CAT-like activity of RuC_6_H_6_, RuC_6_H_6_-PPh_3_, and RuC_6_H_6_-(PPh_3_)_2_ (*n* = 3 independent experiments, data are presented as mean ± SD). In (E), (F), and (I), a.u. indicates the arbitrary units.

Following the observed differences in ROS scavenging performance between the 2 arene ruthenium complexes, we conducted theoretical analyses to elucidate the molecular origins of their distinct biocatalytic activities. Given the superior CAT-like activity of RuC_6_H_6_ over RuC_5_H_5_, we focused on understanding the mechanistic basis for H_2_O_2_ decomposition. Effective catalysis requires both accessibility of the metal center and facile ligand exchange. As shown in Fig. 3A and B, computational analysis revealed that RuC_6_H_6_ undergoes spontaneous hydrolysis in aqueous solution, with a hydrolysis energy of –1.08 eV, which was also confirmed by ^1^H NMR (Fig. S10). In contrast, RuC_5_H_5_ presents a substantially higher energy barrier (5.25 eV), rendering it largely inert to hydrolysis under comparable conditions. Structurally, RuC_6_H_6_ adopts a dichloro-bridged dimeric arrangement in the solid state. Upon aqueous dissolution, these chloride bridges cleave and are replaced by 1 to 3 water molecules [39], creating labile coordination sites that facilitate subsequent H_2_O_2_ binding and activation. Conversely, the ruthenium center in RuC_5_H_5_ is stabilized by 2 aromatic ligands, resulting in a high energy barrier for ligand exchange. This structural rigidity impedes both solvent displacement and exposure of the catalytic site, thereby limiting its catalytic function.

To elucidate the corresponding CAT-like catalytic pathways, we performed systematic DFT calculations. As depicted in Fig. 3C, the initial step is the precatalyst activation from rapid ligand exchange between H_2_O and H_2_O_2_. Then, one H_2_O_2_* dissociates into H_2_O and O*, and the H_2_O leaves. Subsequently, Ru absorbs another H_2_O_2_ molecule, which decomposes into H* and OOH*. The H* species is then bound to O*, resulting in the formation of OH* + OOH* absorbed on the Ru site. The fourth step is that the adsorbed OH* species combines with an H atom to form H₂O, which subsequently desorbs from the surface, leaving behind OO*. Finally, the OO* species desorbs to form O_2_. The corresponding free energy of the above CAT-like path is displayed in Fig. 3D. It is found that Stage III is the rate-determining step (RDS), and the free energy barrier is 0.53 eV. Furthermore, the SOD-like catalytic pathways are depicted in Fig. S11. The SOD-like catalytic cycle of RuC_6_H_6_ involves the sequential adsorption of 2 *OOH species on the Ru site, followed by proton transfer and disproportionation to generate H_2_O_2_ and O_2_. The free energy barrier of the RDS is 0.41 eV, and the overall reaction follows 2OOH → H_2_O_2_ + O_2_.

The catalytic pathways for H_2_O_2_ decomposition were further verified by experiments. As shown in Fig. 3E, UV–Vis spectroscopy was used to monitor the absorbance changes over 4 min during the reaction of materials with H_2_O_2_ in PBS. There were 4 main peaks at 393, 311, 250, and 210 nm, which were assigned to the Ru d-d transition, LMCT, d(Ru) → π*(L), and benzene π→π* transition, respectively [40,41]. Notable changes were observed at 250 and 311 nm, with enhanced absorbance and slight red shifts. These phenomena may be attributed to ligand exchange and metal electron transfer occurring during the reaction. Moreover, the intermediates formed during the reaction were probed using in situ FTIR spectroscopy. It demonstrated the production of ·OOH (1,215 cm^−1^) and ·O_2_^−^ (1,146 cm^−1^) and O_2_ (867 cm^−1^) species during the decomposition of H_2_O_2_, mirroring the DFT-calculated H-transfer process (Fig. 3F) [42–44]. The generation of intermediates was further intuitively visualized by the contour-color-fill graph (Fig. 3G).

To further probe the functional role of labile chloride ligands in H_2_O_2_ catalysis, we introduced triphenylphosphine (PPh_3_), a strong electron-donating ligand with high coordination affinity, into the RuC_6_H_6_ complex to systematically replace the chloride ligands. Under controlled reaction conditions, we successfully synthesized mono- and di-substituted analogs, RuC_6_H_6_-(PPh_3_) and RuC_6_H_6_-(PPh_3_)_2_, respectively (Schemes S2 and S3 and Figs. S12 to S17). CAT-like activity assays revealed a pronounced decrease in H_2_O_2_ decomposition efficiency with increasing PPh_3_ substitution. Within 10 min, unmodified RuC_6_H_6_ decomposed 96.74% of H_2_O_2_, whereas the mono- and di-substituted analogs decomposed only 56.98% and 1.58%, respectively (Fig. 3H and I). This marked reduction in activity is attributed to the occupation of coordination sites by PPh_3_, which impedes ligand exchange with H_2_O_2_. Additionally, steric shielding from the bulky phosphine groups likely limits substrate access to the ruthenium center. These findings underscore the essential role of labile chloride ligands in maintaining pseudo-vacant coordination sites, thereby enabling efficient H_2_O_2_ binding and catalytic turnover.

### Synthesis and characterization of Lipid-Ru

**Fig. 4.**
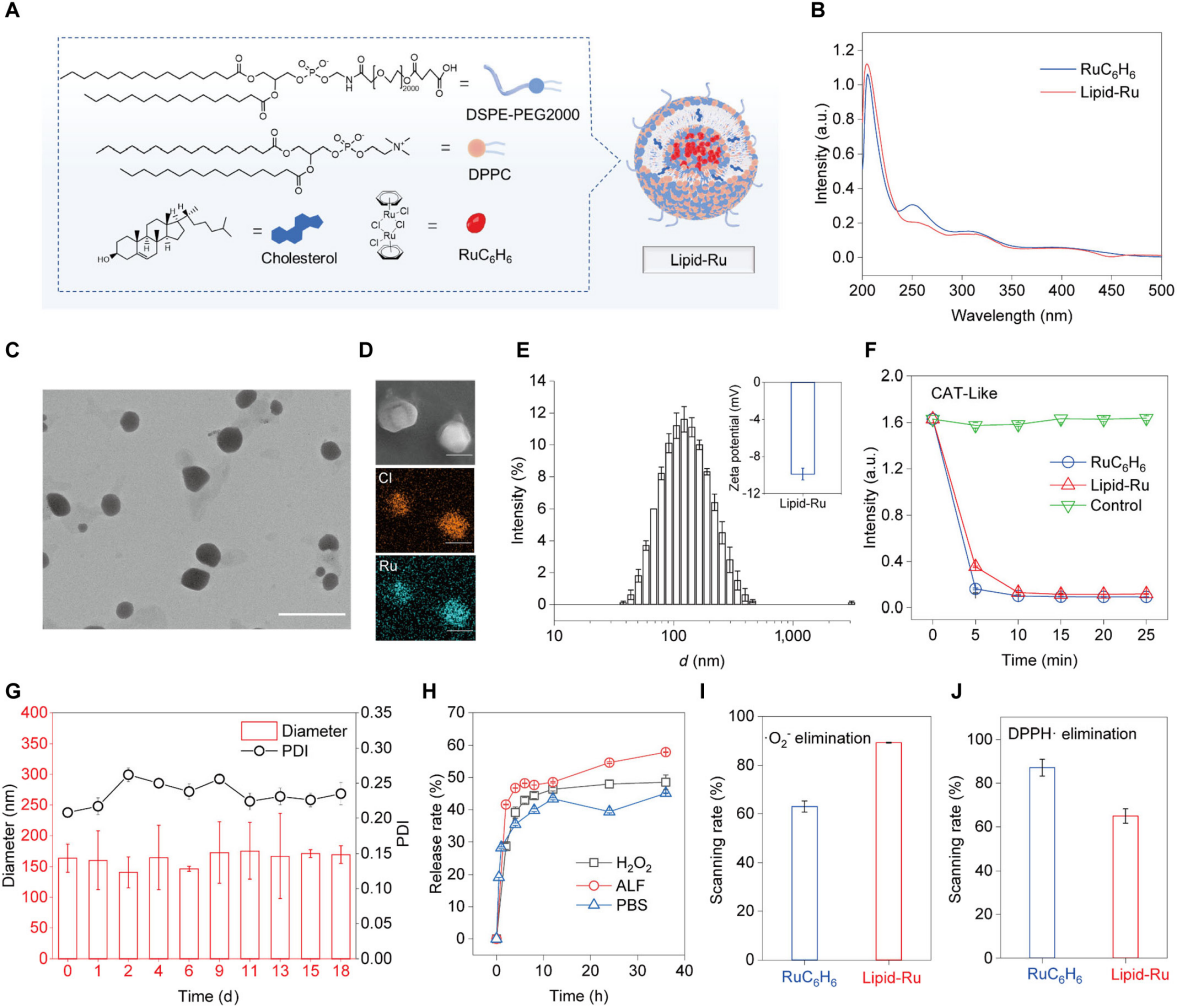
Synthesis and characterization of Lipid-Ru. (A) Illustrating image for the preparation of Lipid-Ru. (B) UV–Vis spectra of RuC_6_H_6_ and Lipid-Ru in water. (C) TEM image of Lipid-Ru; scale bar, 500 nm. (D) The EDS mapping images of Ru, Cl of Lipid-Ru; scale bar, 200 nm. (E) ζ potentials and hydrodynamic size distribution. (F). The dynamic H_2_O_2_ elimination activities occur at different times. (G) Changes of diameters and ζ potential of Lipid-Ru after different storage times at 4 °C. (H) The kinetics of RuC_6_H_6_ release from Lipid-Ru in a PBS, 1 mM H_2_O_2_, and ALF solution by using a dialysis bag at 37 °C. (I) SOD-like property of RuC_6_H_6_ and Lipid-Ru for scavenging of •O_2_^−^ and (J) DPPH• scavenging activity of RuC_6_H_6_ and Lipid-Ru. In (E) to (J), *n* = 3 independent experiments, data are presented as mean ± SD. a.u. indicates the arbitrary units.

To mitigate the potential off-target toxicity of free ruthenium complexes while preserving their anti-ROS activity, we encapsulated RuC_6_H_6_ within liposomal nanocarriers to enhance biocompatibility and optimize pharmacokinetic profiles. As illustrated in Fig. 4A, liposomes were prepared using a thin-film hydration method with a lipid composition comprising 1,2-dipalmitoyl-sn-glycero-3-phosphocholine, cholesterol, and DSPE-PEG2000, resulting in the formation of Lipid-Ru [45,46]. The fluorescent tracer (DiI) was co-incorporated to enable visualization of liposomal localization. UV–Vis absorption spectroscopy confirmed successful encapsulation, with Lipid-Ru exhibiting characteristic absorption peaks of RuC_6_H_6_ at approximately 250, 311, and 393 nm (Fig. 4B). Transmission electron microscopy (TEM) imaging revealed that the resulting nanoparticles adopted a uniform spherical morphology with well-defined structural integrity (Fig. 4C and Fig. S18).

Energy-dispersive x-ray spectroscopy (EDS) confirmed the presence of ruthenium and chlorine within Lipid-Ru, further verifying successful encapsulation of the RuC_6_H_6_ complex (Fig. 4D). Dynamic light scattering measurements indicated an average hydrodynamic diameter of 120 nm and a ζ potential of –9.9 mV (Fig. 4E). Using inductively coupled plasma optical emission spectrometry, the encapsulation efficiency and drug loading rate of RuC_6_H_6_ were determined to be 4.49% and 1.71%, respectively. Lipid-Ru also exhibited excellent colloidal stability, with no significant change in size after 18 days of storage in PBS (Fig. 4G). The release profile of RuC_6_H_6_ from the nanoliposomes was monitored in PBS, 1 mM H_2_O_2_, and artificial lysosomal fluid (ALF) at 37 °C. Approximately 40% of the loaded complex was released within the first 12 h, followed by a more gradual release over the subsequent 28 h (Fig. 4H). The initial rapid release is attributed to the dissolution of drugs adsorbed on the surface and the initial permeability of the membrane, followed by a slow release controlled by membrane diffusion. At 36 h, compared with 45.1% in PBS, the release rate in H_2_O_2_ and ALF increased slightly to 48.5% and 57.9%, respectively. The release data demonstrate that while pathological microenvironmental factors (H₂O₂ and acidic pH) marginally accelerate drug release, the overall release profile remains gradual and sustained. Such a release pattern is expected to facilitate cellular uptake and support sustained intracellular ROS scavenging [47]. Notably, Lipid-Ru largely preserved the intrinsic catalytic activity of free RuC_6_H_6_, maintaining high CAT-like performance as well as the ability to efficiently scavenge •O_2_^−^ and DPPH• radicals (Fig. 4F, I, and J and Fig. S19).

### In vitro biocompatibility, ROS scavenging, and anti-inflammatory performances

**Fig. 5.**
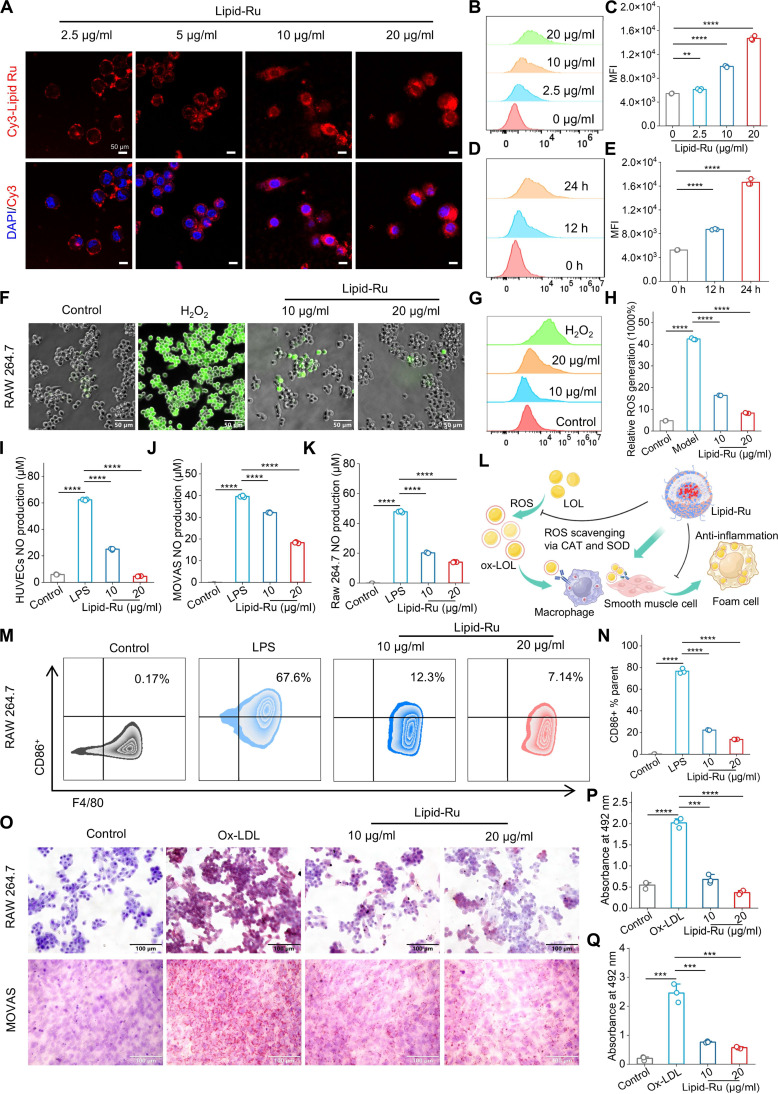
In vitro analysis of ROS-scavenging capability, anti-inflammatory efficacy, macrophage polarization, and foam cell formation after Lipid-Ru treatment. (A) CLSM images, (B) flow cytometry, and (C) mean fluorescence intensity of Cy3 staining with different concentrations of Lipid-Ru. Scale bar, 10 μm. (D) Flow cytometry and (E) mean fluorescence intensity of Cy3 staining at different treatment times. (F) Fluorescence images, Control (Raw264.7+PBS), (G) flow cytometry, and (H) mean fluorescence intensity of DCFH-DA staining. The NO content of (I) HUVECs, (J) MOVAs, (K) RAW264.7 treated with LPS, 10 and 20 μg/ml Lipid-Ru tested by using the Griess assay. (L) Schematic illustration of Lipid-Ru-mediated inhibition of foam cell formation via ROS scavenging in macrophages and smooth muscle cells. (M) Flow cytometry and (N) quantified data of CD86+/F4/80+. (O) Optical microscopy images showing oxLDL-induced foam cell formation in macrophages and MOVAS cells. Scale bars, 100 μm. Quantified contents of Oil Red O (ORO) in foam cells derived from RAW264.7 (P) and MOVAS (Q) cells. In (A) to (Q), *n* = 3 independent experiments, data are presented as mean ± SD. A 2-sided Student *t* test was used to calculate the statistical difference between the 2 groups. Statistical significance was set at **P < 0.01, ***P < 0.001, ****P < 0.0001.

We evaluated the biomedical potential of Lipid-Ru by examining its biocompatibility and cellular uptake. Cytocompatibility was assessed in macrophages (RAW264.7), mouse aortic vascular smooth muscle cells (MOVAS), and human umbilical vein endothelial cells (HUVECs) using the Cell Counting Kit-8 assay. Lipid-Ru showed excellent biocompatibility, maintaining cell viability above 80% at concentrations up to 40 μg/ml (Fig. S20). To determine whether Lipid-Ru can effectively enter cells, a prerequisite for mediating intracellular anti-inflammatory and antioxidant effects [48], we labeled the liposomal membrane with the lipophilic fluorescent dye Cy3 and stained nuclei with 4′,6-diamidino-2-phenylindole. Confocal microscopy in RAW264.7 cells revealed a concentration-dependent increase in red fluorescence, indicating efficient internalization (Fig. 5A). Flow cytometry further confirmed dose-dependent cellular uptake (Fig. 5B and C), as well as a time-dependent increase in fluorescence signal over the incubation period (Fig. 5D and E). These results demonstrate that Lipid-Ru is efficiently internalized in a concentration- and time-dependent manner, fulfilling a key requirement for its potential application in anti-restenosis therapy.

The atherosclerotic plaque microenvironment is characterized by elevated levels of ROS, which contribute to oxidative injury and perpetuate local inflammation [49]. To evaluate the antioxidant and anti-inflammatory potential of Lipid-Ru, we employed H_2_O_2_-stimulated RAW264.7 macrophages as an in vitro model system. Intracellular ROS levels were monitored using the fluorescent probe 2′,7′-dichlorodihydrofluorescein diacetate (DCFH-DA). Both inverted fluorescence microscopy (Fig. 5F) and flow cytometry (Fig. 5G and H) revealed intense green fluorescence in H_2_O_2_-treated cells, indicative of elevated oxidative stress. Notably, Lipid-Ru treatment substantially attenuated ROS generation, with the 20 μg/ml dose reducing intracellular ROS levels to one-fifth of those observed in the H_2_O_2_-only group. These results demonstrate the efficient ROS-scavenging capacity of Lipid-Ru in a living cellular context.

Under physiological conditions, nitric oxide (NO) functions as a key neurotransmitter and vasodilator; however, within atherosclerotic plaques, excess NO reacts with ROS to yield peroxynitrite, particularly the superoxide anion. This reactive species inflicts DNA damage, promotes low-density lipoprotein (LDL) oxidation, induces cytotoxicity, and compromises plaque stability [50,51]. To assess the anti-inflammatory effect of Lipid-Ru, we quantified NO secretion using the Griess assay. After establishing a standard curve for NO detection (Fig. S21), we measured NO levels in inflammatory cells following material treatment. As shown in Fig. 5I to K, lipopolysaccharide (LPS) stimulation markedly elevated NO release in RAW264.7 macrophages, MOVAS SMCs, and HUVECs, reflecting a pro-inflammatory state. Lipid-Ru treatment effectively suppressed NO secretion in all 3 cell types, with an efficacy trend consistent with its ROS-scavenging performance. Together, these results demonstrate that Lipid-Ru exhibits robust antioxidant activity alongside significant anti-inflammatory potential.

Macrophage dysregulation is a key driver of vascular restenosis and atherosclerotic progression, particularly the dominance of the M1 phenotype. Classically activated M1 macrophages secrete a range of pro-inflammatory cytokines that amplify local inflammation and contribute to plaque destabilization [49]. To assess whether Lipid-Ru can modulate this process, we examined its effect on macrophage polarization. RAW264.7 cells were stimulated with LPS and subsequently treated with Lipid-Ru, followed by immunostaining and flow cytometric analysis of the M1 marker CD86. LPS stimulation induced a pronounced shift toward the M1 phenotype, with 76.9% of cells identified as CD86^+^/F4/80^+^ (Fig. 5M and N and Fig. S22). This shift was markedly reversed by Lipid-Ru in a dose-dependent manner: treatment with low and high doses reduced the M1 population to 22.0% and 14.0%, respectively. These findings indicate that suppression of pro-inflammatory macrophage polarization is a central mechanism by which Lipid-Ru exerts its anti-inflammatory activity.

Intimal foam cells, derived from macrophages and migrated SMCs that have ingested oxidized LDL (ox-LDL), represent a hallmark of atherosclerotic lesion progression and are closely linked to oxidative stress and inflammatory injury at sites of endothelial dysfunction [52]. To evaluate the capacity of Lipid-Ru to suppress foam cell formation, we stimulated RAW264.7 macrophages and MOVAS SMCs with LPS and exposed them to ox-LDL. Oil Red O staining revealed substantial intracellular lipid accumulation in both cell types within the model group, indicative of robust foam cell formation. Notably, Lipid-Ru treatment markedly reduced ox-LDL uptake in a dose-dependent manner. Both low and high doses suppressed lipid droplet accumulation, with the 20 μg/ml dose exhibiting the strongest inhibitory effect (Fig. 5O to Q). These in vitro results collectively demonstrate that Lipid-Ru effectively attenuates foam cell generation by limiting ox-LDL internalization in both macrophages and SMCs (Fig. 5L).

### In vivo anti-restenosis efficacy of Lipid-Ru

**Fig. 6.**
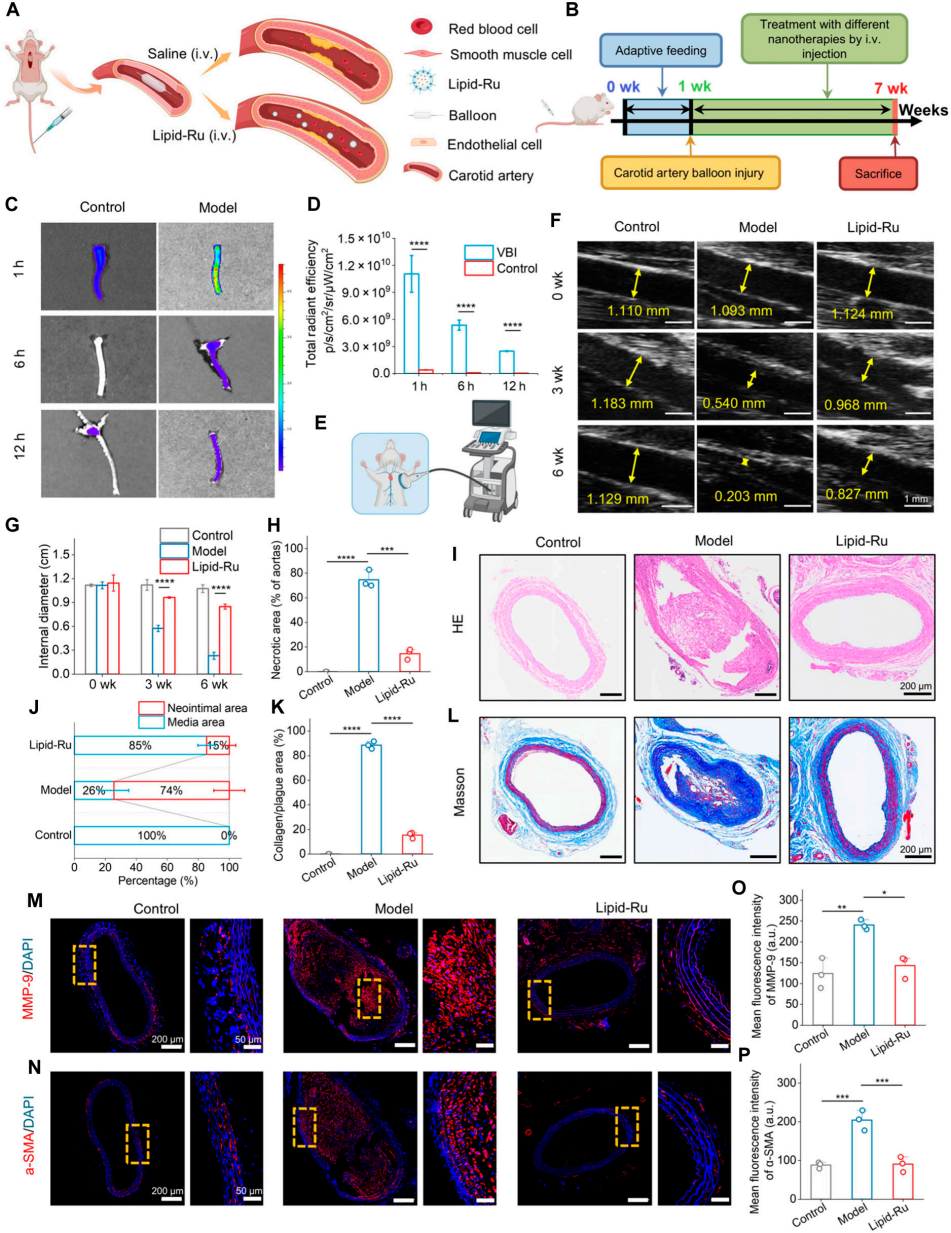
Assessment of the efficacy of Lipid-Ru in preventing restenosis using injured arteries of SD rats. (A) Plaque-bearing SD rats were treated with saline and Lipid-Ru via intravenous (i.v.) administration twice a week for 6 weeks. (B) Schematic of the timeline and treatment protocol for the study. (C) Ex vivo fluorescence images and (D) quantitative data of left common carotid arteries collected at 1, 6, and 12 h after Cy7-labeled Lipid-Ru nanomaterials i.v. administration. (E) Schematic illustration of aortic internal diameter measurement in rats via ultrasound imaging. (F) Ultrasound images and (G) quantitative data of the left common carotid arteries during administration of different reagents at 0, 3, and 6 weeks after surgery. Scale bar, 1 mm. (I) H&E-stained histological sections of the carotid arteries after treatment with different nanomaterials for 6 weeks. Scale bars: 200 μm. Quantitative analysis of the (H) neointima area ratio and (J) neointima/media area ratio measured in HE-stained images from each group. (L) Microscopy images of carotid arteries stained with Masson’s trichrome, and (K) quantitative analysis. CLSM images of carotid arteries stained with (M) anti-MMP-9 antibody and (N) anti-α-SMA antibody. Quantitative analysis of (O) MMP-9 area and (P) α-SMA area in the injury site. In (C) to (P), *n* = 3 independent experiments, data are presented as mean ± SD. A 2-sided Student *t* test was used to calculate the statistical difference between the 2 groups. Statistical significance was set at *P < 0.05, **P < 0.01, ***P < 0.001, ****P < 0.0001. a.u. indicates the arbitrary units.

We next established a rat model of post-angioplasty restenosis to evaluate the in vivo distribution of Lipid-Ru. Two weeks after balloon injury, Cy7-labeled Lipid-Ru was administered intravenously, and its accumulation in the surgically modified carotid artery was monitored using noninvasive imaging. Both in vivo and ex vivo analyses revealed substantial fluorescence signal in the neck region of model rats (Fig. 6A and B and Fig. S23). Time-course imaging showed that fluorescence intensity in the carotid artery of model animals remained strong for up to 12 h, whereas signal in control animals diminished rapidly (Fig. 6C and D). This targeted accumulation can be attributed to vascular endothelial dysfunction following balloon injury, which enhances local vascular permeability and creates an EPR effect. This phenomenon facilitates the passive enrichment of nanoscale agents at sites of vascular remodeling [53].

We next evaluated the therapeutic potential of Lipid-Ru in a rat model of balloon injury-induced restenosis (Fig. 6E) [11]. Treatment was initiated immediately following surgical induction of carotid artery injury. Serial carotid ultrasound imaging at 0, 3, and 6 weeks after intervention revealed a progressive decline in lumen diameter in untreated model animals, whereas Lipid-Ru treatment significantly preserved arterial patency and attenuated the restenosis process (Fig. 6F and G). Histological analysis further supported these findings. Hematoxylin and eosin (H&E) staining of carotid sections (Fig. 6I) demonstrated that Lipid-Ru significantly reduced both the neointimal area and the neointima-to-media ratio compared with the model group (Fig. 6H and J), consistent with the ultrasound observations. Masson’s trichrome staining revealed that Lipid-Ru also reduced collagen deposition within the plaque (Fig. 6K and L), indicating a more stable lesion phenotype. Together, these data suggest that Lipid-Ru mitigates adverse vascular remodeling and plaque progression, thereby lowering the risk of restenosis.

Immunofluorescence analysis was further employed to assess the expression of matrix metalloproteinase-9 (MMP-9) and α-smooth muscle actin (α-SMA) in arterial sections (Fig. 6M and N). Within atherosclerotic plaque, MMP-9, largely secreted by macrophage cells, contributes to plaque instability by degrading extracellular matrix (ECM) components [54,55]. Quantitative evaluation revealed that Lipid-Ru significantly reduced MMP-9 expression in the lesion area (Fig. 6O). Similarly, Lipid-Ru treatment markedly suppressed α-SMA levels compared with the model group, indicating inhibition of vascular SMC proliferation and migration (Fig. 6P). These findings collectively demonstrate that Lipid-Ru enhances plaque stability and mitigates risks of vascular rupture and thrombosis. Furthermore, the Lipid-Ru group exhibited good biocompatibility, with no adverse effects on hematological, hepatic, or renal parameters (Fig. S24). Consistent with this, histopathological evaluation of major organs (heart, liver, spleen, lung, and kidney) revealed no signs of systemic toxicity (Fig. S25). Importantly, Lipid-Ru could be excreted in urine, which supports its favorable clearance profile (Fig. S26).

### In vitro therapeutic mechanisms of Lipid-Ru

**Fig. 7.**
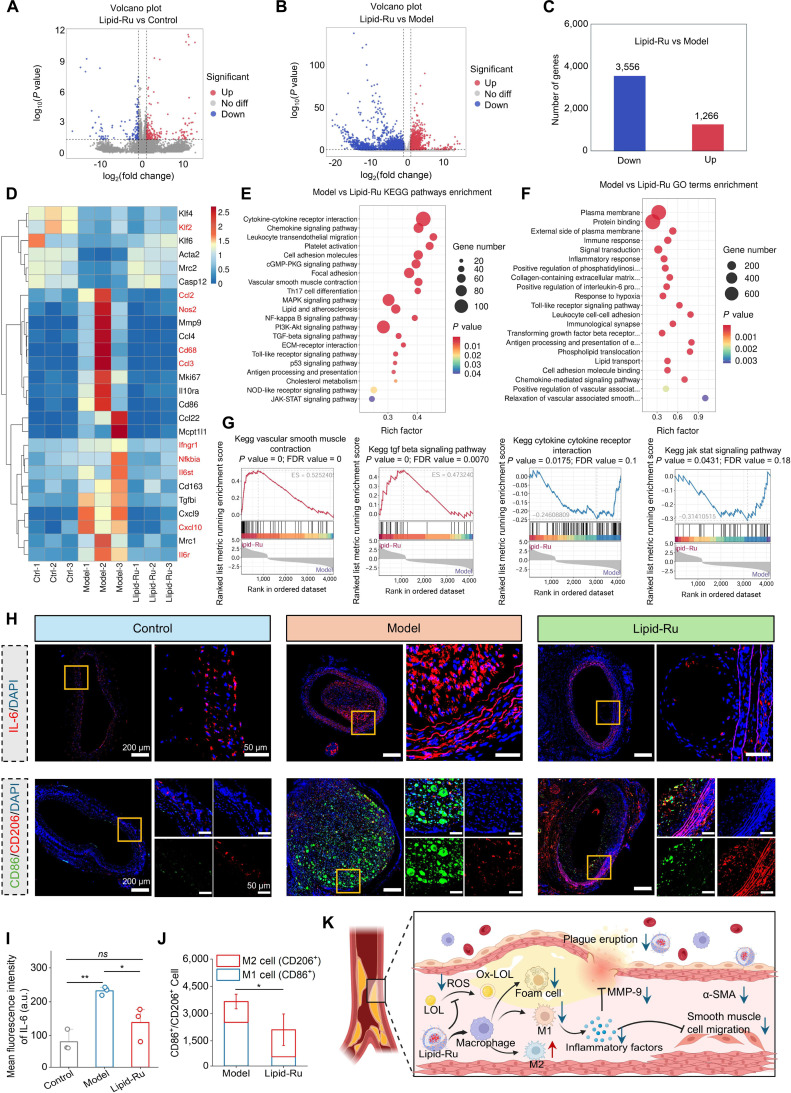
Integrated RNA sequencing and immunofluorescence analysis of carotid artery tissue. (A) Principal component analysis between samples. (B) Differential gene volcano plot and (C) the number of differentially expressed genes in the Lipid-Ru and Model groups. (D) Heat map of differentially expressed genes (red represents genes with relatively high expression levels and blue represents genes with relatively low expression levels). (E) Enriched KEGG pathways of Model versus Lipid-Ru. (F) Enriched GO terms of Model versus Lipid-Ru. (G) GSEA of Model versus Lipid-Ru. NES indicates the normalized enrichment score. (H) Immunofluorescence staining of IL-6 in rats with different treatments. Scale bars, 200 and 50 μm (enlarged image), and immunofluorescence costaining of CD80 and CD206 to observe the macrophage phenotype. Quantitative analysis of fluorescence intensity of (I) IL-6 and (J) CD86^+^/CD206^+^. (K) Schematic illustration of Lipid-Ru for ROS scavenging and preventing restenosis lesions. In (G) to (J), *n* = 3 independent experiments, data are presented as mean ± SD. A 2-sided Student *t* test was used to calculate the statistical difference between the 2 groups. Statistical significance was set at *P < 0.05, **P < 0.01, and ns represents no significant difference.

To gain further insight into the therapeutic mechanism of Lipid-Ru, we performed transcriptomic RNA sequencing on carotid artery tissues. Differential gene expression analysis comparing Lipid-Ru with both Control and Model groups revealed substantial alterations in the transcriptional profile (Fig. 7A to C). Compared with the Model group, Lipid-Ru treatment resulted in the up-regulation of 1,266 genes and down-regulation of 3,556 genes. Hierarchical clustering of these differentially expressed genes showed that Lipid-Ru shifted the transcriptomic signature toward a pattern resembling healthy Ctrl tissue, distinct from the Model profile (Fig. 7D). Notably, Lipid-Ru markedly down-regulated key pro-inflammatory genes, including IL6r, Ccl2, Ccl3, Cxcl10, and CD68, while up-regulating anti-inflammatory mediators such as Klf2, Klf4, and Klf6. This modulation of inflammatory gene expression aligns with the ROS-scavenging capacity observed in cellular assays. Concurrent suppression of Nfkbia, Ifngr1, and NOS2 (iNOS) indicates attenuated inflammatory signaling and reduced NO-related gene expression, consistent with earlier Griess assay results. These findings suggest that Lipid-Ru mitigates inflammation and modulates immune responses by dampening pro-inflammatory pathways while enhancing antioxidant signaling through efficient ROS elimination.

To further delineate the mechanistic basis of Lipid-Ru’s therapeutic effects, we analyzed the top-enriched Kyoto Encyclopedia of Genes and Genomes (KEGG) pathways in the Model versus Lipid-Ru comparison (Fig. 7E). The results indicate that Lipid-Ru down-regulates multiple inflammation-associated signaling cascades, including the Toll-like receptor [56], nucleotide-binding and oligomerization domain (NOD)-like receptor [57], nuclear factor kappa B [58], and janus kinase-signal transducer and activator of transcription (JAK-STAT) pathways [59]. This anti-inflammatory influence was also evident in pathways governing vascular and immune cell activity, including cytokine–cytokine receptor interactions, chemokine signaling, leukocyte transendothelial migration, platelet activation, and cell adhesion. Additionally, Lipid-Ru suppressed the MAPK and p53 signaling pathways, reflecting a reduction in cellular stress and apoptotic signaling [60]. Of particular note was the down-regulation of matrix-degrading enzymes, including MMP-9, within the ECM–receptor interaction pathway, accompanied by decreased expression of focal adhesion-related genes [61]. These alterations suggest an attenuation of ECM degradation, which is a key molecular mechanism contributing to enhanced plaque stability. The broad down-regulation of the comprehensive “Lipid and atherosclerosis” pathway, along with modulated cholesterol metabolism genes, further supports the observed amelioration of lipid accumulation and disease progression.

Furthermore, Lipid-Ru treatment led to the up-regulation of the TGF-β signaling pathway and vascular smooth muscle contraction pathways. TGF-β is a well-characterized anti-inflammatory cytokine that modulates multiple processes in atherosclerosis and restenosis, including inflammation, chemotaxis, fibrosis, proliferation, and apoptosis [57,62]. Previous studies have established that TGF-β activation attenuates atherosclerotic progression by promoting a stable plaque phenotype, in part through enhancing SMC differentiation and inhibiting the transition from a contractile to a synthetic state [63]. The observed up-regulation of vascular smooth muscle contraction pathways further supports this stabilizing effect. Complementary Gene Ontology (GO) enrichment analysis highlighted similar cellular and biological processes (Fig. 7F). Gene Set Enrichment Analysis (GSEA) further confirmed that, compared to the Model group, Lipid-Ru treatment markedly enriched gene signatures related to up-regulated processes, such as TGF-β signaling and vascular smooth muscle contraction, and also down-regulated processes, including cytokine–cytokine receptor interaction and JAK-STAT signaling (Fig. 7G).

Inflammation critically influences macrophage behavior, foam cell formation, and plaque stability throughout atherosclerosis progression. To further validate the anti-inflammatory activity of Lipid-Ru, we performed immunofluorescence analysis of interleukin-6 (IL-6) expression and macrophage polarization in carotid arteries from the restenosis rat model. Lipid-Ru treatment markedly reduced IL-6 levels within carotid plaques (Fig. 7H and I), consistent with in vitro and transcriptomic findings, and confirming suppression of pro-inflammatory signaling as a key mechanism of its therapeutic action. Dual staining for CD86 (M1) and CD206 (M2) markers revealed abundant green fluorescence in model tissues, indicating dominant M1 macrophage infiltration. By contrast, Lipid-Ru treatment substantially reduced green signal intensity while enhancing red CD206 fluorescence, indicating a shift in macrophage polarization toward the anti-inflammatory M2 phenotype (Fig. 7H and J). This repolarization promotes inflammation resolution and attenuates subsequent atherosclerotic complications. Collectively, these results support a multimodal mechanism of action for Lipid-Ru in mitigating post-angioplasty restenosis (Fig. 7K). Through effective ROS scavenging, the material alleviates oxidative stress and inflammatory responses, reverses M1 macrophage polarization, reduces plaque vulnerability, and suppresses neointimal hyperplasia, together contributing to its pronounced therapeutic efficacy.

## Conclusion

Here, we have designed a RuC_6_H_6_-based antioxidant enzyme mimic with pseudo-vacant coordination sites for efficient biocatalytic ROS elimination and attenuation of vascular restenosis. This innovative small-molecular antioxidant enzyme mimic demonstrates potent scavenging capacity against a broad spectrum of ROS, operating through CAT- and SOD-like activities alongside inherent antioxidant properties. Leveraging the EPR effect of liposome encapsulation, Lipid-Ru efficiently localizes to injured vascular regions, where it is further internalized into plaques via macrophage-facilitated transport. Within the inflammatory plaque microenvironment, Lipid-Ru not only neutralizes ROS and suppresses subsequent inflammatory activation but also promotes macrophage polarization toward the anti-inflammatory M2 phenotype, thereby reducing foam cell formation. In a rat model of post-angioplasty restenosis, Lipid-Ru markedly reduced plaque area, inhibited SMC migration, enhanced plaque stability, and alleviated overall macrophage burden and the inflammatory response. Transcriptomic analysis of carotid tissues revealed that these therapeutic benefits are underpinned by down-regulation of key inflammatory and migration-associated pathways, including those linked to smooth muscle proliferation and plaque rupture. In summary, this work establishes Lipid-Ru as an effective ROS-scavenging nanoagent with compelling anti-restenosis performance. More broadly, it provides a conceptual and practical foundation for the rational design of biocatalytic metallodrugs targeting oxidative and inflammatory pathways in diverse pathologies, such as arthritis, diabetic wounds, enteritis, and impaired bone healing.

## Materials and Methods

This section is provided in the Supplementary Materials.

## Data Availability

The data that support the findings of this study are available from the corresponding authors upon reasonable request.
