# Supplementary material for: Bioinspired Ruthenium Arene Complex with Pseudo-Vacant Coordination Sites as Efficient Small-Molecular Antioxidant Enzyme Mimics for Preventing Vascular Restenosis
Source: Research (Wash D C). 2026 Jul 3;9:1353. doi: 10.34133/research.1353 (PMC13329158; doi:10.34133/research.1353)
Supplement: Supplementary 1 — Figs. S1 to S26 Table S1 [file research.1353.f1.docx]

**Supplementary Information for**

**Bioinspired Ruthenium Arene Complex with Pseudo-Vacant Coordination Sites as Efficient Small-Molecular Antioxidant Enzyme Mimics for Preventing Vascular Restenosis**

*Haojie Xu,^1,§^ Yichen Cai,^2,3,§^ Wei Geng,^4^ Zhenyu Xing,^4^ Jiayan Zheng,^5^ Xiaolin Wang,^6^ Yang Gao,^1,*^ Qiu Chen,^2,*^ Li Qiu,^1,*^ & Chong Cheng ^4,5*^*

^1^ Department of Medical Ultrasound, West China Hospital, Sichuan University, 610041, Chengdu, China

^2^ Department of Endocrinology, Hospital of Chengdu University of Traditional Chinese Medicine, 610072, Chengdu, China

^3^ Shanghai Sixth People's Hospital Affiliated to Shanghai Jiao Tong University School of Medicine, Shanghai 200233, China

^4^ College of Polymer Science and Engineering, State Key Laboratory of Advanced Polymer Materials, Sichuan University, 610065, Chengdu, China

^5^ Department of Endodontics, Department of Orthodontics, State Key Laboratory of Oral Diseases & National Clinical Research Center for Oral Diseases, West China Hospital of Stomatology, Sichuan University, Chengdu 610041, China

^6^ School of Pharmacy and State Key Laboratory of Quality Research in Chinese Medicine, Macau University of Science and Technology, Macao, 999078 China

^§^ These authors contributed equally to this work.

* Correspondence: yang.gao0917@scu.edu.cn (Y. Gao); chenqiu1005@cdutcm.edu.cn (Q. Chen); qiulihx@scu.edu.cn (L. Qiu); chong.cheng@scu.edu.cn or chong.cheng@fu-berlin.de (C. Cheng)

**Supplementary Experimental Sections**

**1. Materials and Reagents**

The ruthenium (III) chloride hydrate (RuCl_3_·xH_2_O) was obtained from Energy Chemical (Anhui, China). The 1,3-cyclohexadiene, triphenylphosphine, ammonium hexafluorophosphate, 1,2-dipalmitoyl-sn-glycero-3-phosphocholine (DPPC), and 1,2-distearoyl-sn-glycero-3-phosphoe-thanolamine-N-[(polyethylene glycol)−2000] (DSPE-PEG2000) were purchased from Aladdin reagents (Shanghai, China). Cholesterol and bis(cyclopentadienyl)ruthenium (II) were obtained from Macklin (Shanghai, China). Pure water (18.2 MΩ·cm) used in the experiments was produced from a Milli-Q Academic system (Millipore Corp., Billerica, MA, USA). The remaining reagents, unless specified otherwise, were supplied by Aladdin Reagents (Shanghai, China). All the reagents were of analytical grade and were used as received. Oxidized low-density lipoprotein (ox-LDL), DiI-labeled ox-LDL (DiI-ox-LDL), and Oil Red O (ORO) were obtained from Solarbio (Beijing, China). 4’,6-diamidino-2-phenylindole (DAPI), Cell Counting Kit-8 (CCK-8), and Reactive Oxygen Species Assay Kit were purchased from Beyotime Biotechnology (Shanghai, China). Hematoxylin-Eosin and Masson’s Trichrome Stain Kit were bought from Servicebio (Wuhan, China). All reagents were purchased from Sigma-Aldrich unless otherwise indicated. All chemicals and solvents used are analytical grade.

**2. Structural Characterization**

^1^H NMR spectra were measured by an AV III HD 400 MHz NMR spectrometer (Bruker) at room temperature. A dissolved oxygen meter (INESA, JPSJ-605F) was used to calculate the O_2_ concentration. The UV-vis spectra were acquired using a spectrophotometer (Shimadzu UV-1750). Absorbance was measured using a multi-functional enzyme labeling instrument (ReadMax1900). The inductively coupled plasma optical emission spectrometer (ICP-OES, Thermo ICAP PRO) was used to analyze the loading rate of RuC_6_H_6_ in liposomes. The release profile of the RuC_6_H_6_ from the liposomes was measured by inductively coupled plasma-mass spectrometry (Agilent 7850 ICP-MS). The morphology and size of liposomes were evaluated using a transmission electron microscope (TEM) with a Talos F200S microscope. Energy-dispersive spectroscopy (EDS) mapping was performed via a Titan Themis 60-300 operated at 200kV. Dynamic light scattering (DLS) and zeta potential measurements were carried out using a Malvern Nano-ZS, providing information on the size distribution and surface charge of the liposomes. In our study, *in-situ* FTIR measurements were conducted using an infrared spectrometer (Thermo Scientific, iS50 FTIR) equipped with an *in-situ* spectrum cell (Shanghai Yuanfang Technology Co., Ltd., SPECEL-III).

**3. Density Functional Theory (DFT) Calculation**

The first-principle calculations are performed using the CP2K/Quickstep package^[66]^. The hybrid Gaussian and plane wave (GPW) basis sets and Goedecker–Teter–Hutter (GTH) pseudopotentials are used, with Gaussian functions consisting of a double-ζ plus polarization (DZVP-MOLOPT-GTH) basis. The Perdew–Burke–Ernzerhof (PBE)^[67]^ functional was applied for the structural optimization. All calculations were carried out in the gas phase (vacuum conditions) without the inclusion of implicit or explicit solvation models. The cutoff energy was 400 Ry, and all structures were relaxed until the total energies converged to within 0.001 eV per atom. The van der Waals correction is considered using the Grimme approach (D3) to account for van der Waals interactions within the system.^[68]^

To explore the catalytic effect, the change of Gibbs free energy (ΔG) was calculated, which is defined as:

$$\text{∆G }\text{= ∆E + ∆ZPE +}\text{ ∆H}_{\text{0→298K}}\text{- T∆S}$$

Where ΔE is the energy change obtained from DFT calculations; ΔZPE, ΔH, and ΔS denote the difference in zero-point energy, enthalpy, and entropy due to the reaction, respectively.

**4. Cell Lines and Animals**

Mouse macrophage cell line (RAW264.7), mouse aortic vascular smooth muscle cell line (MOVAS), and human umbilical vein endothelial cell line (HUVEC) were purchased from American Type Culture Collection (USA). All cell lines were checked for cell viability, isozyme detection, DNA fingerprinting, and mycoplasma detection. Male Sprague‒Dawley (SD) rats (200-250 g) were purchased from Huafukang Biological Technology Co. Ltd. (China) and housed in standard rat cages under controlled temperature (23 ± 2 ℃) and humidity (5-60%) with ad libitum access to water and regular rodent food. All animal care and experiments were approved by the Animal Ethics Review Committee of West China Hospital, Sichuan University (No.20220224101). Before further experiments, all rats were acclimatized for at least 7 days.

**5. Experimental Procedures**

**5.1. Synthesis of RuC_6_H_6_**


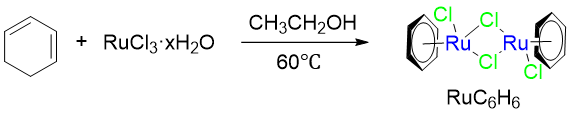


**Scheme S1.** Synthetic scheme for the RuC_6_H_6_.

RuC_6_H_6_ was synthesized according to the reported literature ^[69]^. RuCl_3_·xH_2_O (100 mg, 0.48 mmol) in 20 mL ethanol was heated under reflux with 1 mL 1,3-cyclohexadiene. After 3 h, the orange-brown solid, which had precipitated, was centrifuged, washed with ethanol, and dried in vacuo overnight. Yield: 68 mg, 57%. ^1^H NMR (400 MHz, DMSO-d_6_) δ 5.97 (s, 6H). ^13^C NMR (100 MHz, DMSO-d_6_) δ 88.26.

**5.2. RuC_6_H_6_-(PPh_3_) and RuC_6_H_6_-(PPh_3_)_2_**


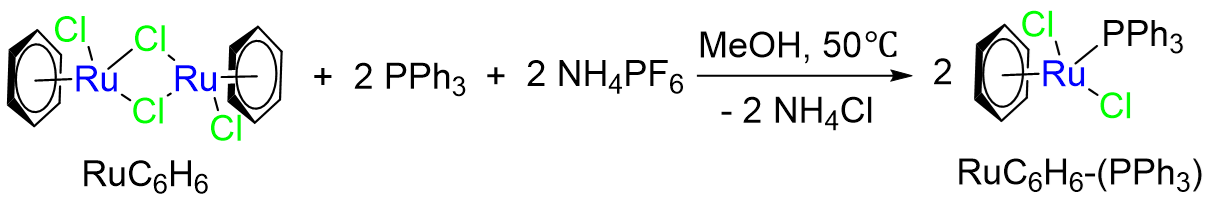


**Scheme S2.** Synthetic scheme for the RuC_6_H_6_-(PPh_3_).


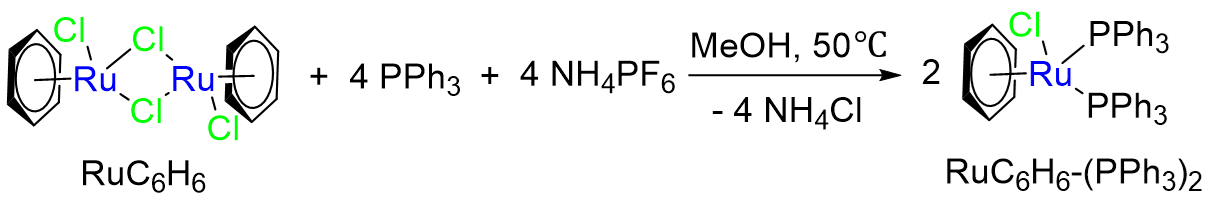


**Scheme S3.** Synthetic scheme for the RuC_6_H_6_-(PPh_3_)_2_.

The synthesis of RuC_6_H_6_-(PPh_3_) and RuC_6_H_6_-(PPh_3_)_2_ was carried out as follows^[70]^: RuC_6_H_6_ (50 mg, 0.1 mmol) was dissolved in 10 mL of methanol. To this solution, two equivalents and four equivalents of triphenylphosphine (tpp) and NH_4_PF_6_ were added, respectively. The reaction mixture was stirred at 50 ℃ under a nitrogen atmosphere for 3 hours, yielding RuC_6_H_6_-(PPh_3_) and RuC_6_H_6_-(PPh_3_)_2_. After the reaction, the solution was concentrated by rotary evaporation. The crude product was precipitated by adding twice the volume of diethyl ether, followed by filtration and washing with diethyl ether. The solid was then redissolved in a small amount of acetone, and insoluble impurities were removed by filtration. The product was reprecipitated by adding an excess of diethyl ether to the filtrate. Finally, the solid was collected and dried under vacuum.

RuC_6_H_6_-(PPh_3_), Yield: 50 %. ^1^H NMR (400 MHz, DMSO-d_6_) δ 5.40 (s, 6H), 7.26-7.44 (m, 9H), 7.72-7.77 (t,6H). ^13^C NMR (100 MHz, DMSO-d_6_) δ 134.40 (d, J_C,P_=9.0 Hz), 133.81 (d, J_C,P_=50.0 Hz) 130.74, 128.48 (d, J_C,P_=9.0 Hz), 89.32 (d, J_C,P_=3.0 Hz). ^31^P NMR (162 MHz, DMSO-d_6_) δ 27.49.

RuC_6_H_6_-(PPh_3_)_2_ Yield: 42 %. ^1^H NMR (400 MHz, CD_3_Cl) δ 5.56 (s, 6H), 7.28-7.29 (m, 12H), 7.37-7.42 (m, 18H). ^13^C NMR (100 MHz, CD_3_Cl) δ 133.91(t, J_C,P_=4.5 Hz), 133.60 (m, J_C,P_=24.0 Hz, overlapped with signal at 133.91), 131.05, 128.59 (t, J_C,P_=5.0 Hz), 96.64 (t, J_C,P_=2.5). ^31^P NMR (162 MHz, CD_3_Cl) δ 20.50.

**5.3. Synthesis of Lipid-Ru**

Liposomes were prepared as described earlier with minor modification^[71,72]^. Briefly, DPPC, DSPE-PEG2000, cholesterol, and Dil (molar ratio 49:15:35:1) were dissolved in CHCl_3_ in a round-bottom flask. After solvent evaporation, the lipid film was rehydrated with a 2 mg/mL RuC_6_H_6_ solution dissolved in 5 mL PBS (pH=7.4). Subsequently, the resulting liposomes were obtained using a probe sonication method (power level, 300 W; duration, 5 min) in an ice bath. To remove unencapsulated RuC_6_H_6_, the liposome suspension was dialyzed against PBS (pH 7.4) using a dialysis membrane with a molecular weight cutoff (MWCO) of 3500 Da. Dialysis was performed at 4 ℃ for 24 hours. The purified liposomes were collected by centrifugation, resuspended in PBS, and stored at 4 ℃ until further use. The encapsulation efficiency and drug loading of RuC_6_H_6_ were determined by ICP-OES.

**6. In Vitro Drug Release Study**

The drug release profile of RuC_6_H_6_-loaded liposomes was evaluated using the dialysis bag method. Briefly, 2 mL of the liposome suspension was placed in a dialysis bag (molecular weight cutoff 3500 Da), which was then immersed in PBS (pH 7.4), 1 mM H_2_O_2_, and artificial lysosomal fluid (ALF). ALF was prepared according to the previous literature^[73]^. The release study was conducted under constant stirring at 100 rpm and a temperature of 37 ℃ to simulate physiological conditions. At predetermined time intervals (0, 0.5, 1, 4, 8, 12, 24, 36 hours), 1 mL of the external medium was withdrawn and replaced with an equal volume of fresh medium to maintain a constant volume. The concentration of released RuC_6_H_6_ in the collected samples was quantified using ICP-MS. The cumulative drug release percentage was calculated and plotted as a function of time to generate the release profile.

**7. ROS Scavenging Activity Tests**

**7.1．CAT-like Tests-H_2_O_2_ Elimination**

A total of 10 mM of H_2_O_2_ and 0.1 mM of biocatalysts were mixed in PBS (pH=7.4) to 2 mL. Then, 100 μL of the above solution was mixed with a Ti(SO_4_)_2_ solution (100 μL, 13.9 mM), and the absorbance value at 405 nm was recorded every 5 minutes for 30 minutes. After the reaction reached 30 min, the absorbance of the solution at 405 nm was tested to evaluate the H_2_O_2_ scavenging ability of the biocatalysts. The CAT-like activity at different pH levels was tested with 25 μM of biocatalysts.

**7.2．CAT-like Tests-O****_2_ Generation Assay**

A reaction mixture containing 200 mM H_2_O_2_ and biocatalysts at concentrations of 2.5, 3.75, 5, and 10 μM was prepared in 20 mL of PBS (pH = 7.4). The oxygen (O_2_) concentration was monitored using a dissolved oxygen meter (INESA, JPSJ-605F), with measurements recorded every 5 s until 300 s. To analyze the biocatalytic kinetics of O_2_ generation, 20 μM of biocatalysts and different concentrations of H_2_O_2_ (50, 100, 200, 250, 300, 400, 450, 500, and 600 mM, respectively) were mixed in PBS to obtain 20 mL, and then the O_2_ concentration was measured every 5 s until 125 s. The reaction rates were plotted against their corresponding H_2_O_2_ concentration and then fitted with the Michaelis–Menten curves (Equation (1)). Furthermore, a linear double-reciprocal plot (Lineweaver–Burk plot, Equation (2)) was used to determine the maximal reaction velocity (*V_max_*) and Michaelis constant (*K_m_*). Additionally, the turnover number (TON, the maximum number of substrates converted per unit active catalytic center) was calculated using Equation (3). The [S] is the concentration of H_2_O_2_, and [E_0_] represents the molar concentration of metal in materials.

$V=\frac{V_{max}\times[S]}{K_{m}+[S]}$ (S1)

$\frac{1}{V}=\frac{K_{m}}{V_{max}}\times\frac{1}{[S]}+\frac{1}{V_{max}}$ (S2)

$TON=\frac{V_{max}}{[E_{0}]}$ (S3)

Among them, *V_max_* is the maximum reaction rate in the ROS catalytic reaction; *K_m_* interprets the affinity between the catalyst and the substrate (the smaller the value, the better the affinity); and TON reflects the maximum number of converted substrates per unit active catalytic atom, which interprets the intrinsic activity independent of the amount of active center.

**7.3．•O_2_^−^ Scavenging Test**

1 mg KO_2_ was dissolved into 1 mL dimethyl sulfoxide solution (DMSO, containing 3 mg/mL 18-crown-6-ether) to generate and stabilize •O_2_^−^. Then, the RuC_6_H_6_ and RuC_5_H_5_ were dispersed into the above KO_2_/DMSO solution at a final concentration of 100 μM. After reaction for 5 min, the remnant •O_2_^−^ will be trapped by nitroblue tetrazolium (NBT)-DMSO solution (10 μL, 10 mg/mL). The absorbance of the solution at 680 nm was measured and then compared with the original concentration of •O_2_^−^ to ensure the •O_2_^−^ scavenging ability.

**7.4．•O_2_^−^ Scavenging Test**

A total of 50 μg mL^-1^ 2,2-diphenyl-1-picrylhydrazyl (DPPH•, Aladdin, Shanghai, China) ethanol solution and biocatalysts (100 μM) were mixed to form a 2 mL solution. Then, the mixture was placed in the dark for reaction, and the absorbance of the solution at 519 nm was measured after 30 min. The DPPH• radical scavenging abilities were calculated.

**8. UV-vis Spectra Change during H_2_O_2_ Elimination**

A 1 mL PBS solution (pH = 7.4) containing 100 μM of the sample was prepared and added to a quartz cuvette. After stabilization, the initial spectrum was recorded. Subsequently, 10 mM H_2_O_2_ was rapidly introduced into the cuvette, and UV-vis measurements were performed immediately. Absorption spectra were recorded at 40-second intervals until the reaction concluded at 4 minutes.

**9. Details for *in-situ* Fourier Transform Infrared (FTIR) Measurement**

In our study, *in-situ* FTIR measurements were conducted using an infrared spectrometer (Thermo Scientific, iS50 FTIR) equipped with an *in-situ* spectrum cell (Shanghai Yuanfang Technology Co., Ltd., SPECEL-III). We typically prepare a Nafion solution consisting of 210 µL of isopropyl alcohol, 750 µL of deionized water, and 40 µL of Nafion (Perfluorosulfonic acid ion exchange resin, Energy Chemical, 5% w/w in water and 1-propanol). This is followed by the formation of a catalyst/Nafion solution at a concentration of 10 mg/mL. During the experiment, we deposited 40 µL of the catalyst solution onto a ZnSe crystal surface, allowed it to dry, and then installed the crystal into the *in-situ* cell. Subsequently, 5 mL of a 0.5 M H_2_O_2_ solution was added to the *in-situ* cell, and we collected the *in-situ* FTIR spectra at specific intervals, maintaining a reaction time of 10 minutes.

**10. Cell Culture**

Tissue culture flasks or plastic dishes were used to maintain cells in an incubator under a humidified atmosphere at 37 °C with 5% CO_2_. Human umbilical vein endothelial cells (HUVECs) and mouse vascular smooth muscle cells (MOVAS) were cultured in Dulbecco's modified Eagle's medium (DMEM, Gibco, USA), and RAW 264.7 murine-derived macrophages were incubated in Roswell Park Memorial Institute (RPMI) 1640 medium supplemented with 10% fetal bovine serum (FBS, Gibco, USA) and 1% penicillin/streptomycin antibiotics. The culture media were replaced every 48 h for HUVECs and MOVAS cells and every 24 h for Raw264.7 cells.

**11. CCK-8 Cytotoxicity of Nanomedicines**

Cell viability was determined by a cell counting kit-8 (CCK-8) (Beyotime Biotechnology, China) assay following the manufacturer’s instructions. Briefly, HUVECs, RAW 246.7 cells, and MOVAS cells were seeded into 96-well plates at densities of 1 × 10^4^, 5 × 10^4^, and 1 × 10^4^ per well, respectively, for 24 h. Subsequently, fresh culture medium containing different concentrations of Lipid-Ru (0, 2.5, 5, 10, 20, 40 µg/mL) was replaced. After 24 h of incubation, the culture medium was removed, the cells were rinsed, and serum-free medium with 10% CCK-8 solution was added. After incubation for another 1 h in an incubator, a spectrophotometer (Thermo Fisher Scientific, USA) was used to measure the optical absorbance density (OD) at 450 nm. Cells that received no Lipid-Ru were used as controls. The cell viability rate (%) was calculated by normalizing the mean OD value to that in the control group.

**12.** **Cellular Uptake Observation by Fluorescence Microscopy**

**12.1. Observation by Fluorescence Microscopy**

Raw 264.7 cells were seeded into 24-well plates at a density of 1 × 10^5^ cells per well in 1 mL of growth medium for 12 h. Then, the culture medium was removed, and 1 mL of fresh medium with 100 ng/mL lipopolysaccharide (LPS, L2880, Sigma, USA) was added. After pretreatment at 37 °C for 2 h, different concentrations of Cy3-labeled Lipid-Ru (2.5, 5, 10, 20 µg/mL) were added. After incubation at 37 °C for 24 h, the cells were rinsed with PBS and further stained with DAPI (P0131, Beyotime Biotechnology, China) according to the operation instructions. Subsequently, fluorescence images were obtained by confocal laser scanning microscopy (CLSM, Olympus, Japan).

**12.2. Quantification by Flow Cytometry**

Raw 264.7 cells were cultured in 6-well plates at a density of 1 × 10^6^ cells per well in 1 mL of growth medium. After 12 hours of incubation, the culture medium was replaced with 1 mL of fresh medium containing LPS at 100 ng/mL, and the culture was incubated for an additional 2 hours. Then, the cells were cultured with Cy3-labeled Lipid-Ru at 10 µg/mL for various periods (0, 12, and 24 h) at 37 °C. Finally, the cells were washed, digested, and centrifuged for flow cytometric analysis using a Cytoflex (Beckman Coulter, USA). Using similar methods, dose-dependent internalization profiles (2.5, 5, 10, 20 µg/mL) were examined after 24 h of incubation.

**13. Intracellular ROS Generation in Macrophages**

RAW246.7 cells were cultured at a density of 1 × 10^6^ and 5 × 10^5^ per well, in 6-well plates and 12-well plates, respectively, for 24 h. Subsequently, cells treated with fresh medium served as controls, while those stimulated with 100 ng/mL H_2_O_2_ alone were used as a model. Cells coincubated with H_2_O_2_ and various concentrations of Lipid-Ru (10, 20 µg/mL) were used as treatment groups. After incubation for 4 h, the medium was removed, and the Reactive Oxygen Species Assay Kit (S0033M, Beyotime Biotechnology, China) was used to probe intracellular ROS. Thirty minutes after cultivation at 37 °C in the dark, the adherent cells were gently washed twice, and fluorescence images were acquired immediately using a fluorescence microscope (IX83, Olympus, Japan). Using similar procedures, fluorescent signals were quantified by flow cytometry after digestion and harvest of DCFH-DA-stained cells.

**14. In Vitro Intracellular NO Detection**

HUVECs, MOVAs, and RAW264.7 cells were cultured in 12-well plates and incubated for 12 h at 37 °C. The experiment was divided into four groups: cells treated with fresh culture medium served as the control group; cells incubated with 100 ng/mL LPS and 100 IU/mL γ-interferon (IFN-γ) for 24 hours served as the model group; cells incubated with 100 ng/mL LPS, 100 IU/mL IFN-γ, and 10 μg/mL Lipid-Ru or 20 μg/mL Lipid-Ru for 24 hours served as the low-dose and high-dose groups, respectively. Cells from each group were lysed using Cell and Tissue Lysis Buffer (for Nitric Oxide Assay) (S3090, Beyotime Biotechnology, China). The nitric oxide (NO) content was measured by detecting the absorbance at 540 nm using a microplate reader.

**15. Flow Cytometry Analysis of Macrophage Polarization.**

RAW246.7 cells were cultured in 6-well plates at a density of 8 × 10^5^ per well for 24 h. Subsequently, cells treated with fresh medium served as a control, while those stimulated with 100 ng/mL H_2_O_2_ alone were used as a model. Cells coincubated with H_2_O_2_ and various concentrations of Lipid-Ru (10, 20 µg/mL) were used as treatment groups. After 24 hours of incubation, cells were collected. The medium was aspirated, and 2 mL of PBS was added to each well. The cells were centrifuged at 1500 rpm for 5 minutes and washed twice. The cell count was adjusted to 1×10⁷ cells per flow cytometry tube, and 1 μL of Fc receptor blocking agent was added. After vortexing, the cells were incubated at 4 °C for 20 minutes. Surface staining was performed by adding CD86 antibody / F4/80 antibody / no antibody (prepared by diluting 1 μL of antibody in 1 mL BD Brilliant Stain Buffer at a 50:1 dilution ratio), followed by vortexing and incubation at 4°C for 20 minutes. After staining, the cells were centrifuged and washed once or twice. For fixation, 1 mL of fixation/permeabilization working solution was added to each tube to resuspend the cells, followed by light-protected incubation at 4°C for 40–50 minutes. After washing, 350 μL of Perm/Wash Buffer was added, and the cells were placed on ice. Multicolor fluorescence signals were immediately analyzed using a Cytek full-spectrum flow cytometer and sorted, with subsequent data analysis performed using FlowJo software. All antibodies used were from BD Pharmingen™ flow cytometry antibodies.

**16. Treatment Effect of Lipid-Ru on Foam Cell Formation**

RAW 246.7 and MOVAS cells were cultured in 24-well plates at densities of 1 × 10^5^ and 5 × 10^4^ per well, respectively, for 24 h. Subsequently, cells treated with fresh medium were used as controls, stimulated with 100 ng/mL LPS and 50 µg/mL oxidized low-density lipoprotein (ox-LDL, IO300, Solarbio, China) alone as a model, and coincubated with LPS, ox-LDL, and various concentrations of Lipid-Ru (10, 20 µg/mL) as treatment groups. After incubation for 24 h, the cells were washed with PBS and stained with Oil Red O Stain Kit, for Cultured Cells (G1262, Solarbio, China). Finally, the cells were observed using bright-field optical microscopy (IX83, Olympus, Japan). In addition, the concentration of intracellular ORO was measured by the absorbance at 492 nm via UV‒visible spectrometry (Thermo Fisher Scientific, USA) after extraction with pure isopropanol.

**17. Establishing a Vascular Balloon Injury (VBI) Model**

A carotid artery balloon injury model was generated according to the methods described by D.A. Tulis^[74]^. Male Sprague-Dawley (SD) rats aged 6 weeks, weighing 200 ± 20 g, were acclimatized for 1 week before the modeling surgery. Under gas anesthesia using isoflurane, the left common carotid artery (LCCA) was exposed. A 2F Fogarty balloon catheter (Baxter Edwards Healthcare Corp., Irvine, CA, USA) was inserted into the LCCA, the balloon was inflated, and the catheter was withdrawn and reinserted four times to frictionally denude the endothelium. Sham-operated rats underwent the same surgical procedure without catheter insertion and withdrawal. All rats were housed in an animal facility with controlled temperature and humidity under a 12-hour light/12-hour dark cycle. Animals had free access to food and water, and all were reared identically. Healthy male SD rats were randomly divided into a Control group, a Lipid-Ru + Model (Lipid-Ru group), and a Model + saline group (Model group). After the modeling surgery, the Model group received intravenous tail vein injections of saline, while the Lipid-Ru group received intravenous injections of Lipid-Ru (0.5 mg/kg) twice weekly for 6 weeks.

**18. Assessment of Antiatherogenic Efficacy**

At 0, 3, and 6 weeks post-modeling surgery, non-invasive ultrasound examinations were performed on the injured carotid arteries of rats from different groups to assess restenosis at the modeling site. At week 2 post-modeling surgery, rats from the Model and Control groups were intravenously injected with Cy7-labeled Lipid-Ru. Using an *in vivo* imaging system, the accumulation of Lipid-Ru at the local modeling site was observed in vivo at 0, 6, and 12 hours post-injection. Subsequently, rats were euthanized at 0, 6, and 12 hours post-injection for *ex vivo* observation, and the carotid arteries were isolated to examine the accumulation of Lipid-Ru on the inner wall of the injured carotid artery. At 6 weeks post-modeling surgery, rats were euthanized, and the left common carotid arteries were harvested for histopathological analysis, including hematoxylin and eosin (H&E) staining, Masson's trichrome staining, and immunofluorescence staining. Major organs, including the heart, liver, spleen, lung, and kidney, were harvested for histopathological analysis (H&E staining).

**19. *In Vivo*** **Serum Safety Assessment**

The experiment concluded on the 14th day after administration in each group of rat. The blood samples were collected. A portion of the samples was placed in vacuum anticoagulant tubes for the detection and analysis of routine blood parameters at the end of the experiment. The other portion was placed in regular centrifuge tubes and allowed to stand at 4 °C. After the samples had separated, they were kept at room temperature for several minutes and then centrifuged at 3000 rpm for 15 min in a refrigerated centrifuge to separate and collect the serum. Subsequently, blood biochemical indicators were analyzed using an automatic biochemical analyzer (BC-2800Vet, Mindray Global, China).

**20. Inductively Coupled Plasma Mass Spectrometry (ICP-MS) Detection**

To determine the metabolic pathways of nanoparticles, primary metabolites, such as urine and feces, were collected from rats in each group after 2 weeks of administration. The samples were then subjected to microwave digestion. The Ru amounts were quantified using an inductively coupled plasma mass spectrometer (Agilent 7850).

**21. *In Vivo* Fluorescence Imaging of Lipid-Ru**

To monitor the accumulation of Lipid-Ru in the LCCA after the modeling surgery in vivo, SD rats weighing 200 ± 12 g received a single tail vein intravenous injection of Cy7-labeled Lipid-Ru (2 mg/kg) post-surgery. In vivo fluorescence imaging was performed at 0, 6, and 12 hours after Lipid-Ru injection using a PerkinElmer Spectrum IVIS (USA). Subsequently, rats were euthanized, and the LCCA from the modeled injury site was harvested for ex vivo fluorescence imaging using the same instrument.

**22. *In Vivo* Carotid Artery Ultrasound Measurement**

To visualize the degree of restenosis in the injured carotid artery, a Vevo 3100 Imaging System (Fujifilm VisualSonics, Toronto, Canada) with a 40-MHz linear array ultrasound transducer (MX550D, VisualSonics Inc., Canada) was used. In vivo vascular ultrasound examination of the LCCA was performed. Samples were coded with random numbers before analysis. The researchers performing the measurements were unaware of the group identities until after all data were collected. During examination, rats were anesthetized with isoflurane, and the LCCA was examined longitudinally. Morphological changes of the injured common carotid artery were observed consecutively at 0, 3, and 6 weeks post-balloon injury (VBI). The minimum internal diameter of the injured artery was measured.

**23. Transcriptome sequencing and data analysis**

In our experimental protocol, a cDNA library constructed using technology from pooled RNA from carotid tissue samples of SD rats was sequenced on the Illumina NovaseqTM 6000 sequence platform. Using the Illumina paired-end RNA-seq approach, we sequenced the transcriptome, generating a total of million 2 x 150 bp paired-end reads. Total RNA was extracted using Trizol reagent (Thermofisher, 15596018) following the manufacturer's procedure. The total RNA quantity and purity were analyzed using the Bioanalyzer 2100 and RNA 6000 Nano LabChip Kit (Agilent, CA, USA, 5067-1511). High-quality RNA samples with RIN number > 7.0 were used to construct the sequencing library. Genes differential expression analysis was performed by DESeq2 software between two different groups (and by edgeR between two samples). The genes with the parameter of false discovery rate (FDR) below 0.05 and absolute fold change ≥ 2 were considered differentially expressed genes. Differentially expressed genes were then subjected to enrichment analysis of GO functions and KEGG pathways. We conducted comprehensive bioinformatics analyses through the OmicStudio tools at https://www.omicstudio.cn/tool, encompassing GO term enrichment, PCA, KEGG classification and pathway enrichment, and GSEA.

**24. Statistical analysis**

Statistical analysis was performed using GraphPad Prism 8.0 software (GraphPad Software Inc.), and figures were generated with Origin 2022b. Image analysis for both in vitro and in vivo experiments was conducted using ImageJ software. Experimental parameters, including sample size (n), statistical significance (p), data normalization protocols, and specific statistical tests, are comprehensively detailed in the corresponding figure legends. Quantitative data were collected at least three independent times. All data are expressed as the mean values ± SD. Statistical significance was calculated using the two-sided Student’s t-test or one-way analysis of variance (ANOVA); all tests were two-sided. Statistical significance was set at **p*<0.05, ***p*<0.01, ****p*<0.001, *****p*<0.0001, and ns represents no significant difference.

**Supplementary Figures**

**Figure S1.** ^1^H NMR spectrum (400 MHz, DMSO-d_6_, 298 K) of RuC_6_H_6_.

**Figure S2.** ^13^C NMR spectrum (100 MHz, DMSO-d_6_, 298 K) of RuC_6_H_6_.

**Figure S3.** FTIR patterns of RuC_6_H_6_ and cyclohexadiene.

**Figure S4.** The dynamic H_2_O_2_ elimination activities at different times.

**Figure S5.** The H_2_O_2_ elimination rate at 5 min for different concentrations.

**Figure S6.** The H_2_O_2_ elimination rate at 10 min at different time points after exposed in nature light.

**Figure S7.** The H_2_O_2_ concentration at 5 and 10 minutes at different pH levels.

**Figure S8.** The dynamic O_2_ generation property during 125 s at different H_2_O_2_ concentrations.

a

b

**Figure S9.** a) UV-Vis spectrum of ABTS^+•^ treated with RuC_5_H_5_ and RuC_6_H_6_; b) Scanvenging rate of ABTS^+•^ after treated with RuC_5_H_5_ and RuC_6_H_6_ at 10 min.

**Figure S10.** ^1^H NMR spectrum (400 MHz, D_2_O, 298 K) of RuC_6_H_6_ disolved in D_2_O. The three sets of aromatic proton signals correspond to three distinct mono-nuclear aquated species, δ 5.90 [RuC_6_H_6_Cl_2_(D_2_O)], δ 5.98 [RuC_6_H_6_Cl(D_2_O)_2_]^+^, δ 6.08 [RuC_6_H_6_(D_2_O)_3_]^2+^, which arise from the stepwise replacement of chloride ligands by water molecules.


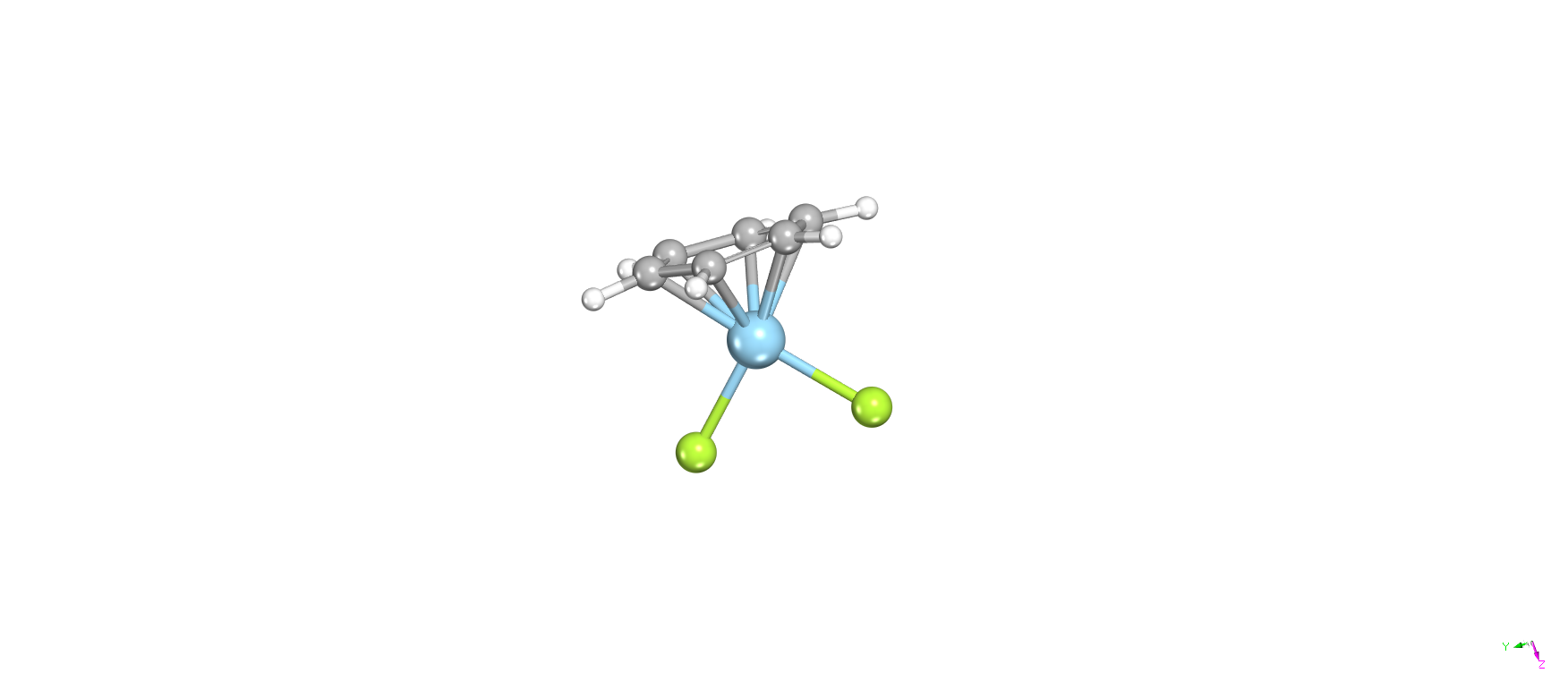

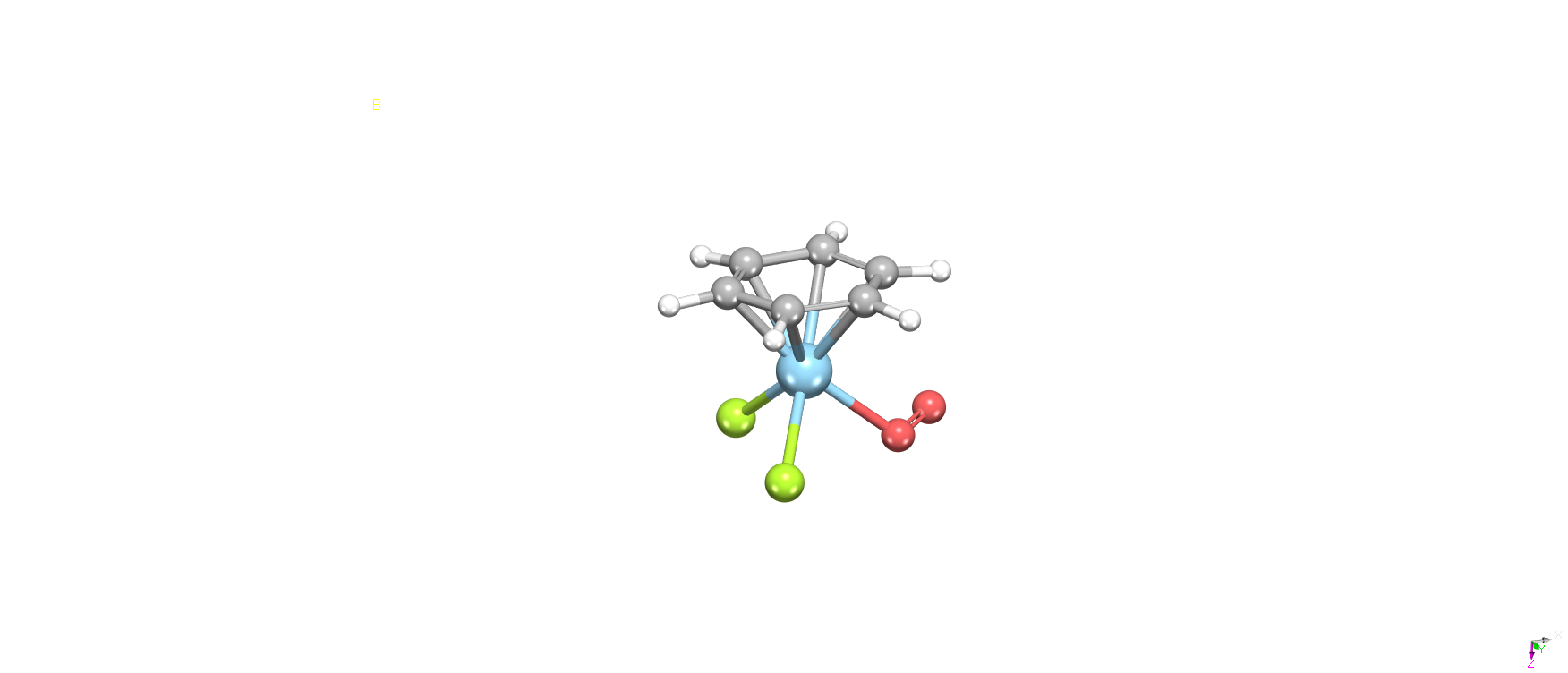

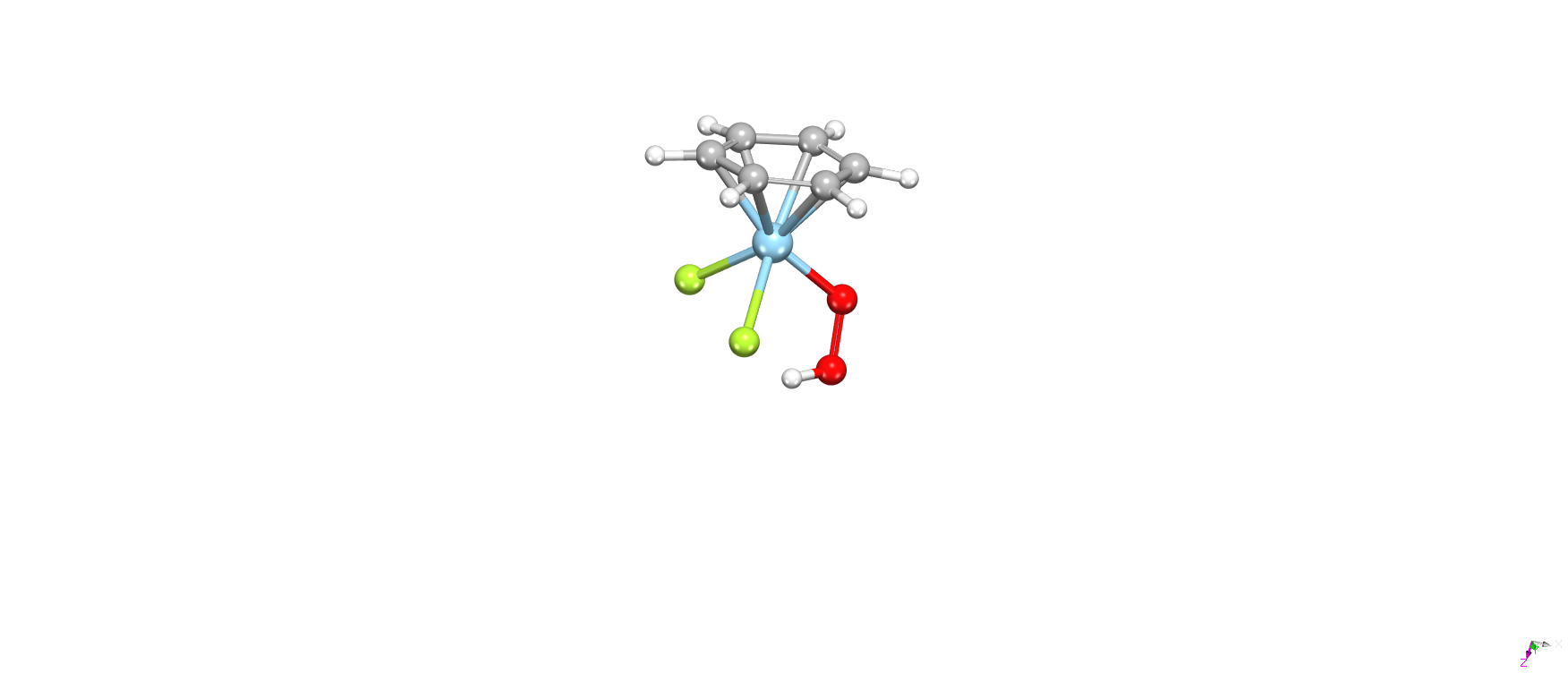


a

b

**Figure S11. a)** Calculated SOD-like catalytic pathways, and b) corresponding free energy profiles.

**Figure S12**. ^1^H NMR spectrum (400 MHz, DMSO-d_6_, 298 K) of RuC_6_H_6_-(PPh_3_).

**Figure S13.** ^13^C NMR spectrum (100 MHz, DMSO-d_6_, 298 K) of RuC_6_H_6_-(PPh_3_).

**Figure S13.** ^31^P NMR spectrum (162 MHz, DMSO-d_6_, 298 K) of RuC_6_H_6_-(PPh_3_).

**Figure S15.** ^1^H NMR spectrum (400 MHz, CD_3_Cl, 298 K) of RuC_6_H_6_-(PPh_3_)_2_.

**Figure S16.** ^13^C NMR spectrum (100 MHz, CD_3_Cl, 298 K) of RuC_6_H_6_-(PPh_3_)_2_.

**Figure S17.** ^31^P NMR spectrum (162 MHz, CD_3_Cl, 298 K) of RuC_6_H_6_-(PPh_3_)_2_.


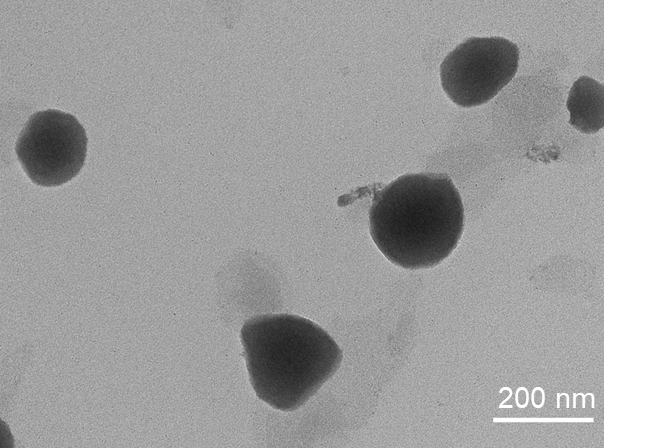

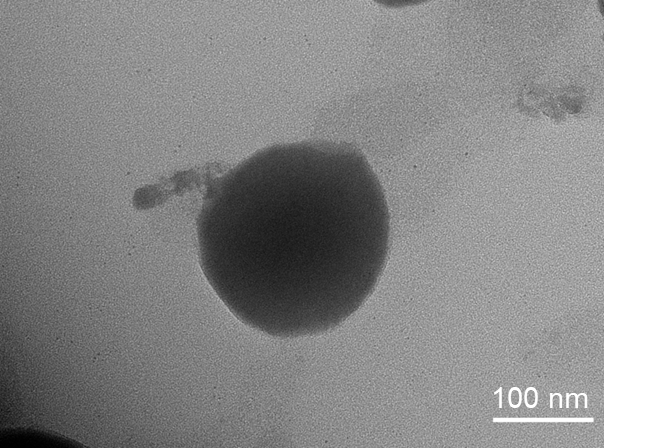


**Figure S18.** TEM images of Lipid-Ru.

**Figure S19.** The dynamic O_2_ generation property of RuC_6_H_6_ and Lipid-Ru at different times.

a

b

c

**Figure S20.** The Cell-Counting-Kit-8 (CCK8) assay of Lipid-Ru toward (a) RAW 264.7 cells, (b) MOVAS, and HUVECS at the predetermined concentration.

**Figure S21.** Standard curve for NO quantification using the Griess assay.


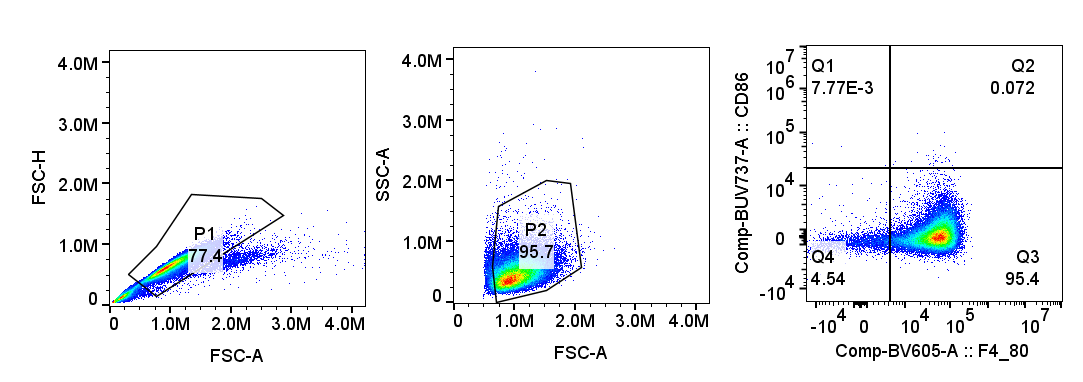


**Figure S22.** A Gating strategy for Fig. 4m.

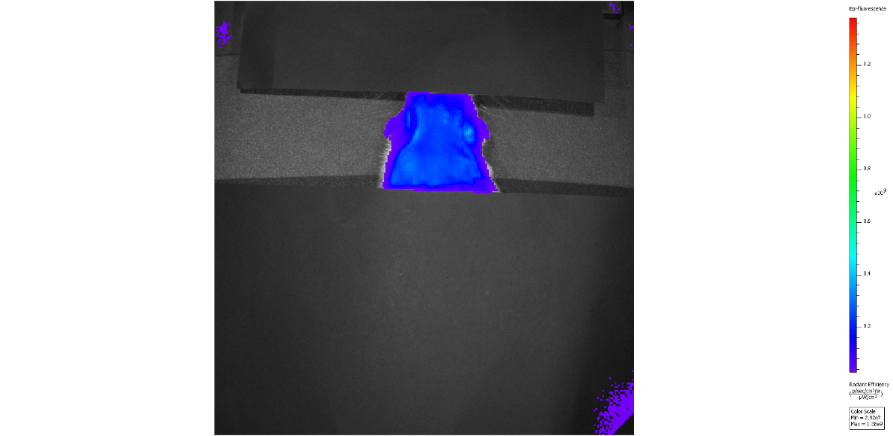

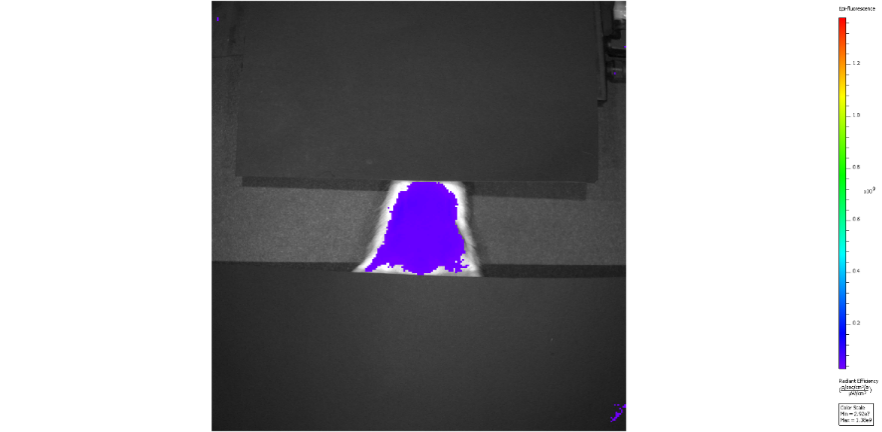

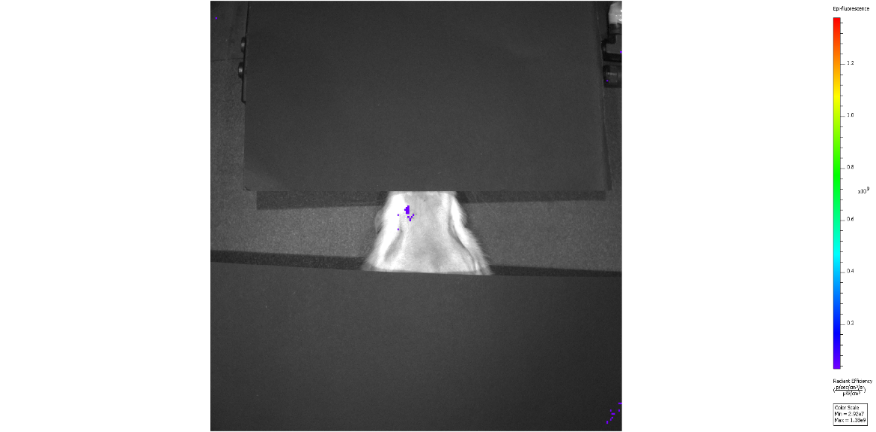

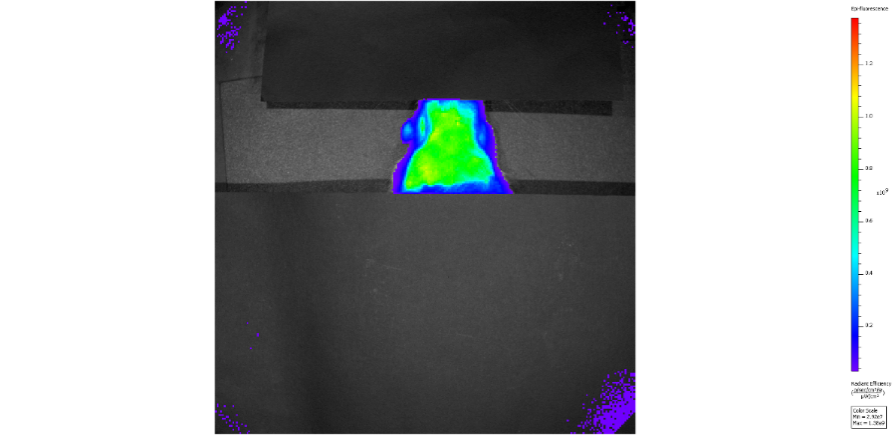

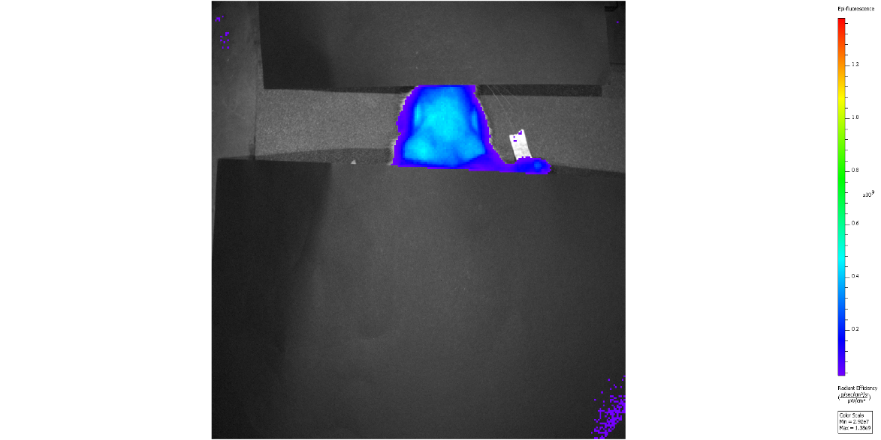

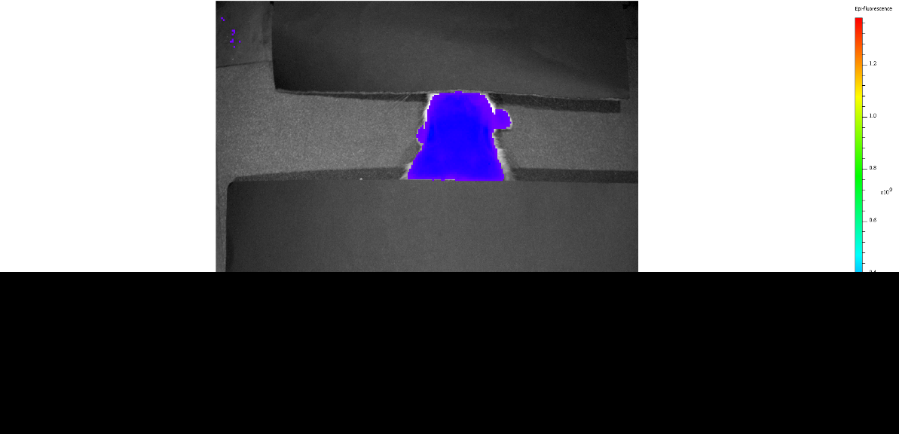


Control

Model

1H

6H

12H


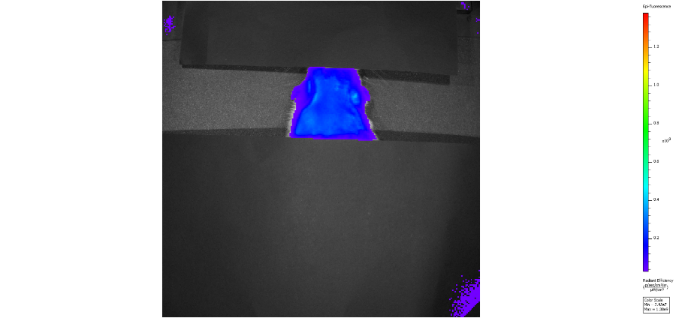


a

b

**Figure S23.** (a) In vivo fluorescence images and (b) quantitative data of a fixed neck region in rats collected at 1, 6, and 12 h after Cy7-labeled Lipid-Ru nanomaterials i.v. Administration.

a

a

a

a

a

b

b

b

b

**Supplementary Figure 24.** a) Blood routine analysis (WBC, white blood cell; Gran, granulocyte Count; Gran (%), granulocyte percentage; RBC, granulocyte percentage; red blood cell; HGB, hemoglobin. b) Blood biochemistry analysis (AST, aspartate aminotransferase; ALT, alanine aminotransferase; UREA, urea nitrogen; CREA, creatinine; n = 3). ns represents no significant difference.


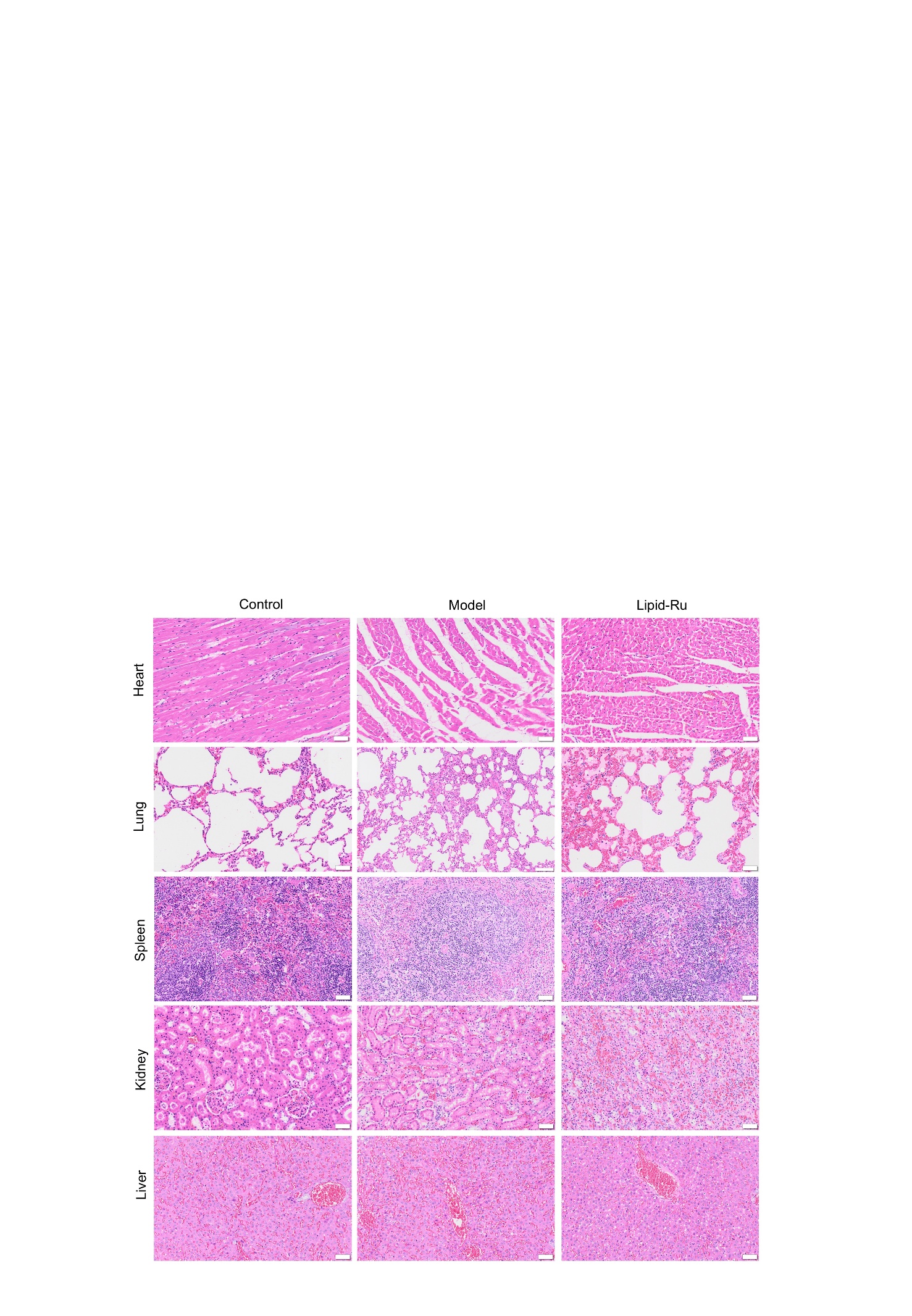


**Figure S25.** H&E staining assay of normal organs of different treatment groups at the end of atherosclerosis therapeutic experiments. Scale bar, 100 µm.

**Figure S26.** The ICP-MS test measured Ru concentrations in rat urine and feces after the injection of the Lipid-Ru for 1 and 14 days.

**Supplementary Tables**

**Table S1.** Comparison of *V_max_* and TON with recently reported state-of-the-art ROS-scavenging biocatalysts. TON=*V_max_*/[E_0_], where [E_0_] is the mole concentration of metal in the whole nanomaterials.

| **Biocatalysts** | ***V*_max_ (μM·s^-1^)** | **[E_0_] (μM)** | **TON (s^-1^)** | **Ref.** |
| --- | --- | --- | --- | --- |
| RuC_6_H_6_ | 23.59 | 13.33 | 1.77 | This work |
| RuTeNRs | 0.98 | 196340 | 5.00E-06 | ^[75]^ |
| MnO_2_ | 2.4 | 4965.52 | 4.83E-04 | ^[76]^ |
| BSA-IrO_2_ | 1.87 | 518.52 | 3.60E-03 | ^[76]^ |
| Co_3_O_4_ NC | 1.23 | 246 | 5.00E-03 | ^[77]^ |
| OxgeMCC-r | 0.2 | 29.28 | 6.83E-03 | ^[78]^ |
| Co_3_O_4_ NR | 1.88 | 250.67 | 7.50E-03 | ^[76]^ |
| Co_3_O_4_ NP | 2.38 | 250.53 | 9.50E-03 | ^[76]^ |
| IrO_x_ NPs | 5.64 | 522.22 | 1.08E-02 | ^[79]^ |
| IrO_x_ | 5.72 | 519.7 | 1.10E-02 | ^[76]^ |
| Pd OA | 5.9 | 234.41 | 2.52E-02 | ^[77]^ |
| Co_3_O_4_ NPs | 11.2 | 248.8 | 4.50E-02 | ^[80]^ |
| Mn_3_O_4_ | 10.73 | 198.7 | 5.40E-02 | ^[81]^ |
| Co_3_O_4_ | 11.55 | 206.25 | 5.60E-02 | ^[81]^ |
| MCCP | 4.75 | 81.66 | 5.82E-02 | ^[76]^ |
| Mn_3_O_4_ Cb | 5.3 | 65.45 | 0.08 | ^[82]^ |
| Mn_3_O_4_ Ph | 5.8 | 65.45 | 8.86E-02 | ^[82]^ |
| CoSe | 7.32 | 72.69 | 0.1 | ^[83]^ |
| Mhp | 7.37 | 65.45 | 0.11 | ^[78]^ |
| MC-1.0 | 24.82 | 118.19 | 0.21 | ^[81]^ |
| Cu NCs | 418.41 | 1820 | 0.23 | ^[84]^ |
| Fe Nzy | 1.22 | 4.82 | 0.25 | ^[85]^ |
| Cu_5.4_O | 3.92 | 15.04 | 0.26 | ^[86]^ |
| Mfk | 21.75 | 65.45 | 0.33 | ^[78]^ |
| Ru_SA_-CN | 10.02 | 13.01 | 0.77 | ^[87]^ |
| Cu_x_O | 109.2 | 125.81 | 0.87 | ^[88]^ |

REFERENCES

[66] J. VandeVondele, M. Krack, F. Mohamed, M. Parrinello, T. Chassaing, J. Hutter, *Comput. Phys. Commun.* **2005**, *167*, 103.

[67] J. P. Perdew, K. Burke, M. Ernzerhof, *Phys. Rev. Lett.* **1996**, *77*, 3865.

[68] S. Grimme, J. Antony, S. Ehrlich, H. Krieg, *J. Chem. Phys.* **2010**, *132*, 154104.

[69] M. A. Bennett, A. K. Smith, *J. Chem. Soc. Dalton Trans.* **1974**, 233.

[70] M. Zander, *Chem. Ber.* **1982**, *115*, 3449.

[71] B. A. Lakshmi, A. S. Reddy, R. Sangubotla, J. W. Hong, S. Kim, *Colloids Surf. B Biointerfaces* **2021**, *204*, 111773.

[72] B. Geers, I. Lentacker, N. N. Sanders, J. Demeester, S. Meairs, S. C. De Smedt, *J. Controlled Release* **2011**, *152*, 249.

[73] T. Jiang, I. Odnevall Wallinder, G. Herting, *Int. Sch. Res. Not.* **2012**, *2012*, 379697.

[74] D. A. Tulis, *Methods Mol. Med.* **2007**, *139*, 1.

[75] S. Kang, Y.-G. Gil, D.-H. Min, H. Jang, *ACS Nano* **2020**, *14*, 4383.

[76] J. Zhou, D. Xu, G. Tian, Q. He, X. Zhang, J. Liao, L. Mei, L. Chen, L. Gao, L. Zhao, G. Yang, W. Yin, G. Nie, Y. Zhao, *J. Am. Chem. Soc.* **2023**, *145*, 4279.

[77] W. Ma, J. Mao, X. Yang, C. Pan, W. Chen, M. Wang, P. Yu, L. Mao, Y. Li, *Chem. Commun.* **2018**, *55*, 159.

[78] D. Wang, H. Wu, S. Z. F. Phua, G. Yang, W. Qi Lim, L. Gu, C. Qian, H. Wang, Z. Guo, H. Chen, Y. Zhao, *Nat. Commun.* **2020**, *11*, 357.

[79] W. Zhen, Y. Liu, W. Wang, M. Zhang, W. Hu, X. Jia, C. Wang, X. Jiang, *Angew. Chem. Int. Ed Engl.* **2020**, *59*, 9491.

[80] J. Mu, L. Zhang, M. Zhao, Y. Wang, *J. Mol. Catal. Chem.* **2013**, *378*, 30.

[81] Q. Tian, W. Wang, L. Cao, X. Tian, G. Tian, M. Chen, L. Ma, X. Liu, Z. Yuan, C. Cheng, Q. Guo, *Adv. Mater.* **2022**, *34*, 2207275.

[82] N. Singh, M. Geethika, S. M. Eswarappa, G. Mugesh, *Chem. Weinh. Bergstr. Ger.* **2018**, *24*, 8393.

[83] Y. Deng, Y. Gao, T. Li, S. Xiao, M. Adeli, R. D. Rodriguez, W. Geng, Q. Chen, C. Cheng, C. Zhao, *ACS Nano* **2023**, *17*, 2943.

[84] C. Liu, Y. Cai, J. Wang, X. Liu, H. Ren, L. Yan, Y. Zhang, S. Yang, J. Guo, A. Liu, *ACS Appl. Mater. Interfaces* **2020**, *12*, 42521.

[85] J. Xi, R. Zhang, L. Wang, W. Xu, Q. Liang, J. Li, J. Jiang, Y. Yang, X. Yan, K. Fan, L. Gao, *Adv. Funct. Mater.* **2021**, *31*, 2007130.

[86] T. Liu, B. Xiao, F. Xiang, J. Tan, Z. Chen, X. Zhang, C. Wu, Z. Mao, G. Luo, X. Chen, J. Deng, *Nat. Commun.* **2020**, *11*, 2788.

[87] Y. Sun, S. Mu, Z. Xing, J. Guo, Z. Wu, F. Yu, M. Bai, X. Han, C. Cheng, L. Ye, *Adv. Mater. Deerfield Beach Fla* **2022**, *34*, e2206208.

[88] C. Hao, A. Qu, L. Xu, M. Sun, H. Zhang, C. Xu, H. Kuang, *J. Am. Chem. Soc.* **2019**, *141*, 1091.
